# Cryopreserved Dental Pulp Tissues of Exfoliated Deciduous Teeth Is a Feasible Stem Cell Resource for Regenerative Medicine

**DOI:** 10.1371/journal.pone.0051777

**Published:** 2012-12-14

**Authors:** Lan Ma, Yusuke Makino, Haruyoshi Yamaza, Kentaro Akiyama, Yoshihiro Hoshino, Guangtai Song, Toshio Kukita, Kazuaki Nonaka, Songtao Shi, Takayoshi Yamaza

**Affiliations:** 1 Department of Molecular Cell Biology and Oral Anatomy, Graduate School of Dental Science, Kyushu University, Fukuoka, Japan; 2 Department of Pediatric Dentistry, Graduate School of Dental Science, Kyushu University, Fukuoka, Japan; 3 Center for Craniofacial Molecular Biology, Herman Ostrow School of Dentistry of USC, University of Southern California, Los Angeles, California, United States of America; 4 Department of Pedodontics, School of Stomatology, Wuhan University, Wuhan, China; Universidade de Sao Paulo, Brazil

## Abstract

Human exfoliated deciduous teeth have been considered to be a promising source for regenerative therapy because they contain unique postnatal stem cells from human exfoliated deciduous teeth (SHED) with self-renewal capacity, multipotency and immunomodulatory function. However preservation technique of deciduous teeth has not been developed. This study aimed to evaluate that cryopreserved dental pulp tissues of human exfoliated deciduous teeth is a retrievable and practical SHED source for cell-based therapy. SHED isolated from the cryopreserved deciduous pulp tissues for over 2 years (25–30 months) (SHED-Cryo) owned similar stem cell properties including clonogenicity, self-renew, stem cell marker expression, multipotency, *in vivo* tissue regenerative capacity and *in vitro* immunomodulatory function to SHED isolated from the fresh tissues (SHED-Fresh). To examine the therapeutic efficacy of SHED-Cryo on immune diseases, SHED-Cryo were intravenously transplanted into systemic lupus erythematosus (SLE) model MRL/*lpr* mice. Systemic SHED-Cryo-transplantation improved SLE-like disorders including short lifespan, elevated autoantibody levels and nephritis-like renal dysfunction. SHED-Cryo amended increased interleukin 17-secreting helper T cells in MRL/*lpr* mice systemically and locally. SHED-Cryo-transplantation was also able to recover osteoporosis bone reduction in long bones of MRL/*lpr* mice. Furthermore, SHED-Cryo-mediated tissue engineering induced bone regeneration in critical calvarial bone-defect sites of immunocompromised mice. The therapeutic efficacy of SHED-Cryo transplantation on immune and skeletal disorders was similar to that of SHED-Fresh. These data suggest that cryopreservation of dental pulp tissues of deciduous teeth provide a suitable and desirable approach for stem cell-based immune therapy and tissue engineering in regenerative medicine.

## Introduction

Mesenchymal stem cells (MSCs) have been isolated from a variety of fetal and adult tissues and considered as an ideal candidate source for cell-based therapy due to their unique properties such as multipotency and immunomodulatory functions [1]. Many researchers have investigated to apply MSCs as progenitors of osteoblasts for bone tissue engineering. Clinical evidences support the efficacy of MSC-based skeletal tissue regeneration [2], [3]. On the other hand, MSCs exert striking regulatory effects on immune cells such as T- and B-lymphocytes, dendritic cells and natural killer cells [4], [5]. This immunological traits of MSCs lead to take clinical advantages to immune diseases such as acute graft-versus-host-disease (GVHD) [4], [6], hematopoietic stem cell (HSC) engraftment [7], [8] and systemic lupus erythematosus (SLE) [9].

Recent discovery has evaluated that fresh dental pulp tissues of human exfoliated deciduous teeth preserve MSC population, termed SHED [10]. SHED display typical stem cell properties including clonogenicity, cell proliferation and multipotency to differentiate into odontoblast/osteoblast-, adipocyte-, and neural cell-like cells [10]. SHED also express a unique *in vivo* tissue regeneration capability of forming dentin/pulp and bone/bone marrow structures when subcutaneously transplanted into immunocompromised mice [10]. SHED implantation govern bone repair in critical-sized bone defects in mouse calvarias [11] and swine mandible [12]. Moreover, systemic SHED-transplantation exhibited effective improvement on SLE-like disorders including hyper-autoantibody levels, renal dysfunction and hyperactivity of interleukin 17 (IL-17)-producing helper T (Th17) cells, in MRL/*lpr* mice [13]. Therefore SHED are considered to be a feasible and promising cell source for cell-based tissue engineering and immune therapy in regenerative medicine.

Exfoliated deciduous teeth possess advantages of minimal invasiveness and easily accessible tissue source in comparison with other human tissues such as bone marrow and adipose tissue [10]. However, the effective preservation of deciduous teeth has remained a primary concern for clinical applications of SHED. In addition, SHED isolation is impractical immediately after the exfoliation of deciduous teeth because the opportunity of the exfoliation is unpredictable. Recently, cryopreservation of human cells and tissues is amenable to be a reliable and feasible approach for stem cell storage [14]. Herein, we cryopreserved dental pulp tissues of exfoliated deciduous teeth for over 2 years and investigated the effects of the long term cryopreservation on the recovering of SHED properties including *in vitro* and *in vivo* biological and immunological properties. Furthermore, we assessed the therapeutic efficacy of the recovered SHED from the cryopreserved deciduous dental pulp tissues on immune modulation and bone regeneration in SLE model-MRL/*lpr* and bone defect model-immunocompromised mice.

## Materials and Methods

### Ethics Statement

Procedures using human samples (exfoliated deciduous teeth and peripheral blood) were conducted in accordance with the Declaration of Helsinki and approved by the Kyushu University Institutional Review Board for Human Genome/Gene Research (Protocol Number: 393-01). We obtained written informed consent from all the children’s parents on the behalf of the children participants involved in this study. All animal experiments were approved by the Institutional Animal Care and Use Committee of Kyushu University (Protocol Number: A21-044-1) and conformed to all the guidelines outlined in the Guide for the Care and Use of Laboratory Animals by the National Institutes of Health (NIH).

### Human Subjects

Human exfoliated deciduous teeth were collected as discarded biological/clinical samples from children (5–7-year-old) at Department of Pediatrics of Kyushu University Hospital. Human peripheral blood mononuclear cells (PBMNCs) were collected from healthy volunteers (28–34 year-old).

### Cryopreservation of Deciduous Dental Pulp Tissues of Exfoliated Deciduous Teeth and Isolation and Culture of SHED

A protocol for the cryopreservation of dental pulp tissues of exfoliated deciduous teeth was summarized in **Figure S1**. Dental pulp tissues were separated from exfoliated deciduous teeth. Half of the samples were mixed with a cryopreserved medium at 4°C and kept overnight at −80°C. The cryopreserved medium consisted of 10% dimethyl sulfoxide (DMSO) (Sigma, St Louis, MO) and 90% fetal bovine serum (FBS) (Equitech-Bio, Kerrville, TX). They were transferred into liquid nitrogen and stored for over 2 years (25–30 months). The other half of fresh deciduous dental pulp tissues were treated to isolate SHED (SHED-Fresh). SHED from the cryopreserved deciduous pulp tissues (SHED-Cryo), as well as SHED-Fresh, were isolated by an adherent colony-forming unit fibroblasts (CFU-F) method [10], [13], [15]. The cryopreserved tissues were quickly thawed at 37°C. Both cryopreserved and fresh dental pulp tissues were digested with 0.3% collagenase type I (Worthington Biochemicals, Lakewood, NJ) and 0.4% dispase II (Sanko Junyaku, Tokyo, Japan) in phosphate buffered saline (PBS, pH 7.4) for 60 min at 37°C. Single-cell suspensions were obtained through a 70-µm-cell strainer (BD Bioscience, San Jones, CA). The cells (1×10^6^) were seeded on T-75 flasks, washed with PBS after 3 hours and cultured at 37°C in 5% CO_2_ with a growth medium containing 15% FBS (Equitech-Bio), 100 µM L-ascorbic acid 2-phosphate (WAKO Pure Chemical, Osaka, Japan), 2 mM L-glutamine (Nacalai Tesque, Kyoto, Japan) and 100 U/ml penicillin and 100 µg/ml streptomycin (Nacalai Tesque) in alpha Modification of Eagle’s Medium (alphaMEM) (Invitrogen, Carlsbad, CA) and subsequently cultured for 14–16 days until obtaining adherent colonies. The adherent colonies-forming cells were recognized as SHED as described before [10], [13]. The colonies-forming cells were passed and sub-cultured in the growth medium. The medium was changed twice a week.

### Antibodies

Antibodies used in this study are summarized in the **Table S1**.

### Mice

C57BL/6J and Balb/cA-nu/nu mice (female, 6 week-old) were purchased from CLEA Japan. (Tokyo, Japan). C57BL/6J MRL/*lpr* mice (female, 6 week-old) were from Japan SLC. (Shizuoka, Japan). They were housed in temperature- and light-controlled environmental conditions with a 12-hour light and dark cycle, and permitted ad *libitum* consumption of water and standard pellet chow.

### Histology of Cryopreserved Dental Pulp Tissues of Exfoliated Deciduous Teeth

Cryopreserved deciduous pulp tissues were fixed with 4% paraformaldehyde (PFA) in PBS and immersed in O.C.T. compound (Sakura Finetek Japan, Tokyo, Japan). The frozen specimens were cut into 6-µm thick sections. Some sections were stained with hematoxylin and eosin (H&E). The others were immunostained with anti-STRO-1 and anti-human CD146 antibodies by using SuperPicture kit (Invitrogen). Subclass-matched antibodies were used for negative controls for immunohistochemistry. The sections were observed under a microscope BIORVO BZ-9000 (Keyence Japan, Osaka, Japan).

### CFU-F Assay

Cells (10×10^3^) isolated from frozen/fresh deciduous dental pulp tissues were seeded on 100-mm culture dishes and cultured in the growth medium for 16 days. The flasks were treated with 4% PFA and 0.1% toluidine blue in PBS for 18 hours. Colonies containing >50 cells were recognized as single colony clusters under a microscope as previously [10], [13]. The numbers of the colonies were counted.

### Population Doubling Assay

Cells were seeded on T-75 culture flasks (BD Bioscience). When the cells reached at sub-confluent condition, they were passed. These steps were repeated until the cells lost dividing capability. The population doubling score was calculated at every passage according to the equation: log_2_ (number of final harvested cells/number of initial seeded cells) and the total scores were determined by adding up each population doubling score in each sample as described in previous reports [10], [13].

### Bromodeoxyuridine (BrdU) Incorporation Assay

Passaged 3 (P3) SHED were seeded at 1×10^3^ per well on 35-mm dishes and cultured in the growth medium for 2 days. BrdU reagent (Invitrogen) was added at 1∶100 in the medium and subsequently cultured additionally for 24 hours. The cells were fixed in 70% ethanol for 15 min, treated with the BrdU staining kit (Invitrogen) and lightly stained with hematoxylin according to the company’s instruction. The stained samples were observed under BIORVO microscope (BZ-9000, Keyence Japan, Osaka, Japan). Seven images were randomly selected to calculate BrdU-positive nuclei number in each sample. Cell proliferation capacity was shown as a percentage of BrdU-positive nuclei over total nucleated cells [13].

### Telomerase Activity Assay

Telomerase activity was measured by a telomere repeat amplification protocol assay using the quantitative telomerase detection kit (Allied Biotech, Inc., Ijamsville, MD) applied with real-time PCR as referred to our previous reports [13]. As positive control, HEK293T cells were used. Some extracts from each cell were heated at 85°C for 10 min and used as negative control samples. The average starting quantity (SQ) of fluorescence units was used to compare the telomerase activity among the samples.

### Flow Cytometric Analysis for SHED

Passaged 3 (P3) SHED were cultured at 50–60% confluent condition. Cells (100×10^3^/100 µl) were stained with primary specific antibodies. Subclass-matched antibodies were used as negative controls. Flow cytometry was analyzed on FACSCalibur flow cytometer (BD Bioscience) [13]. The number (percentage) of positive cells was determined using CellQuest software (BD Bioscience) by comparison with the corresponding control cells stained with the subclass-matched antibody in which a false-positive rate of less than 1% was accepted.

### Immunofluorescence for Cultured Cells

The cells were fixed with 4% PFA in PBS and blocked with PBS containing 10% normal serum matched to the secondary antibodies. The samples were incubated with the specific antibodies to cell surface markers or the subclass-matched antibodies overnight at 4°C and treated with CF 633-conjugated-secondary antibodies (Biotium, Hayward, CA). Finally, they were stained with 4', 6-diamidino-2-phenylindole (DAPI) (Dojindo, Kumamoto, Japan) and observed under the BIORVO microscope (Keyence Japan). Numbers of cells positive to the specific antibodies and nuclei stained with DAPI were counted and shown as a percentage of positive cells over total nucleated cells.

### Semi-quantitative Reverse Transcription Polymerase Chain Reaction (RT-PCR)

Total RNA was isolated from the cultures using TRIzol (Invitrogen) and digested with DNase I. The cDNA was synthesized from 100 ng of total RNA using Revatra Ace (TOYOBO, Osaka, Japan). The specific primer pairs are listed in **Table S2**. PCR was performed using gene specific primers and RT-PCR Quick Taq HS DyeMix (TOYOBO) at 94°C for 2 min for one cycle and then react for 40 cycles with denature at 94°C for 45 sec, annealing at 56°C for 45 sec, extension at 72°C for 60 sec as one cycles, with a final 10-min extension at 72°C. Finally, 5 µl of each amplified PCR product was analyzed by 2% agarose gel electrophoresis and visualized by ethidium bromide staining. The intensity of bands was measured by using Image-J soft ware and normalized to an internal control gene, *glyceraldehyde 3-phosphate dehydrogenase* (*GAPDH*).

### In vitro Multipotent Assay

#### *In vitro* dentinogenic/osteogenic induction assay

SHED (P3, 5×10^3^) were grown on 60-mm dishes in the growth medium until confluent condition and induced with an dentinogenic/osteogenic medium supplemented with 1.8 mM potassium dihydrogen phosphate (Sigma, St. Louis, MO) and 10 nM dexamethasone (Sigma) in the growth medium. The dentinogenic/osteogenic medium were changed twice a week. One week after the induction, dentinogenic/osteogenic markers were analyzed by semi-quantitative RT-PCR and alkaline phosphatase (ALP) activity test [13]. For mineralized nodule assay, the cultures were stained with 1% Alizarin Red-S (Sigma) at 4 weeks post the osteoinduction. The Alizarin red-positive area was analyzed using NIH image software Image-J and shown as a percentage of Alizarin red-positive area over total area [13].

#### *In vitro* chondrogenic induction assay

SHED (P3, 2×10^6^) were aggregated in a 15 mL tube and cultured with Dulbecco’s modified Eagle’s medium (Invitrogen) supplemented with 15% FBS (Equitech-Bio), 2 mM L-glutamine (Nacalai Tesque), 100 µM L-ascorbate-2-phosphate (Wako Pure Chemicals), 2 mM sodium pyruvate (Nacalai Tesque), 1% insulin-transferring-selene mixture (ITS) (BD Biosciences), 100 nM dexamethasone (Sigma), 10 ng/ml transforming growth factor beta_1_ (TGFbeta_1_) (PeproTech, Rocky Hill, NJ) and 100 U/ml penicillin and 100 µg/ml streptomycin (Nacalai Tesque). The chondrogenic medium were changed twice a week. After 3-week induction, chondrocyte-specific genes were analyzed by semi-quantitative RT-PCR.

#### *In vitro* adipogenic induction assay

Cultured SHED (P3, 5×10^3^/dish) were cultured until the confluent condition and induced in an adipogenic medium with the growth medium plus 500 µM isobutyl-methylxanthine (Sigma), 60 µM indomethacin (Sigma), 0.5 µM hydrocortisone (Sigma) and 10 µM insulin (Sigma). After 6-week induction, the cultures were stained with 0.3% Oil red O (Sigma) to detect lipid droplets. The samples stained with Oil red O were treated with isopropanol and the absorbance of the extracts were measured at 520 nm. Adipocyte-specific genes were also analyzed by semi-quantitative RT-PCR.

#### *In vitro* endothelial cell induction assay

P3 SHED (5×10^3^ cells) were seeded on fibronectin-coated 35-mm dishes (BD Biosciences) and cultured by using a commercial available endothelial cell growth media kit, EGM-2, (Lonza, Basel, Switzerland) with 100 U/ml penicillin and 100 µg/ml streptomycin (Nacalai Tesque) according to the manufacture’s instruction. The endothelial growth medium was changed every 2 days. The cultures were fixed 7 days after the induction. Endothelial cell differentiation was determined by immunofluorescence with anti-CD31 and CD34 antibodies.

#### *In vitro* neuronal cell induction assay

P3 SHED (5×10^3^ cells/well) were plated in laminin-coated 35-mm dishes (BD Biosciences) and cultured in a neurogenic medium containing supplemented with 1xN2 supplement (Invitrogen), 10 ng/ml basic fibroblast growth factor (bFGF) (PeproTech), 10 ng/ml epidermal growth factor (EGF) (PeproTech), 100 U/ml penicillin and 100 µg/ml streptomycin (Nacalai Tesque) in Neurobasal A (Invitrogen) for 21 days according to the recent study [10]. The medium was changed with 50% of fresh medium twice a week. Neural cell differentiation was determined by immunofluorescence with anti-*glial fibrillary acidic protein*, anti-neural filament M and anti-betaIII tubulin antibodies.

#### *In vitro* hepatic induction assay

P3 SHED (P3, 5×10^3^) were seeded on fibronectin-coated 35-mm dishes (BD Biosciences) and cultured with Iscove’s modified Dulbecco’s medium (Invitrogen) supplemented with 10 nM dexamethasone (Sigma), 1% ITS (BD Biosciences), 20 ng/ml EGF (PeproTech), 10 ng/ml bFGF (PeproTech), 20 ng/ml hepatocyte growth factor (PeproTech), 20 ng/ml oncostain M (PeproTech), 100 U/ml penicillin and 100 µg/ml streptomycin (Nacalai Tesque) for 4 weeks. Hepatocyte-specific gene albumin was assayed by semi-quantitative RT-PCR.

### In vivo Tissue Regeneration Assay

Xenogenic transplantation was performed as previously [13], [16]. Cultured SHED (P3) in the growth medium (4×10^6^) were mixed with a carrier, hydroxyapatite tricalcium phosphate (HA/TCP) (40 mg, Zimmer Inc., Warsaw, IN). The mixtures were subcutaneously transplanted into the dorsal surface of 8–10-week-old Balb/c *nude*/*nude*. The implants were harvested 8 weeks after the surgery, fixed with 4% PFA and decalcified with 10% EDTA. Frozen sections were cut and used for H&E staining or immunofluorescence observed under a BIORVO microscope (BZ-9000, Keyence Japan).

### In vivo Self-renewal Assay

*In vivo* self-renewal assay with a sequential transplantation was referred to a recent study [17]. SHED (P3) (4×10^6^) were implanted with HA/TCP carrier (40 mg) (Zimmer) into primary Balb/c *nu*/*nu* mice. Eight weeks after the transplantation, the primary implants were harvested and treated with 0.4% dispase II (Sanko Junyaku) for 60 min at 37°C to obtain single cells from the implants. The cells were stained with R-PE-conjugated anti-human CD146 and magnetic-beads-conjugated anti-R-PE antibodies (Miltenyi Biotec, Bergisch Gladbach, Germany) and sorted magnetically to obtain human CD146-positive cells. The purity was confirmed by flow cytometry with anti-human CD146 or anti-mouse CD146 antibody. The sorted cells were seeded at low density and cultured to obtain CFU-F. Expanded CFU-F-forming cells (P1) (4×10^6^) were subcutaneously transplanted with HA/TCP carriers (40 mg) (Zimmer) into secondary immunocompromised mice for 8 weeks and the secondary implants were assayed by H&E staining and immunofluorescence.

### Histological Analysis of Implant Tissues

Implant tissues were fixed with 4% PFA in PBS overnight and decalcified with 5% EDTA solution (pH 7.4). Frozen sections were cut into 8-µm thickness and treated with H&E staining and immunofluorescence with anti-human mitochondria, anti-STRO-1 or anti-human CD146 antibody. The sections were observed under BIORVO microscope (BZ-9000, Keyence Japan). To measure newly-formed area of mineralized tissue, seven fields were randomly selected and the newly-formed area was calculated by NIH image-J software, and shown as a percentage over total tissue area as described before [13], [16].

### Single Colonies-derived Cell Assay

As referred to a previous study [18], cells isolated from cryopreserved deciduous dental pulp tissues were seeded at 1, 2 or 4 cells per well on 24-well multiplates with the growth medium. The wells containing more than two attached cells were excluded from further analysis. The single cell-attached wells were cultured for 14–16 days and obtained CFU-F-forming cells. The single colony-forming cells were used for population doubling, BrdU incorporation and dentinogenic/osteogenic analyses.

### Induction Assay of Th17 cells

Induction of Th17 cells was performed as previously [13]. Human CD4^+^CD25^−^ T cells were magnetically sorted from human PBMNCs using CD4^+^CD25^+^ regulatory T cell isolation kit (Miltenyi Biotec). They (1×10^6^/well) were activated by plate-bounded anti-CD3 (5 µg/ml) and soluble anti-CD28 (1 µg/ml) antibodies for 3 days and loaded on SHED (20×10^3^/well) with recombinant human TGFbeta_1_ (2 µg/ml, PeproTech) and IL-6 (50 µg/ml, PeproTech) for 3.5 days. T cells were stained with anti- PerCP-conjugated CD4 and FITC-conjugated anti-CD8a antibodies, treated with R-PE-conjugated anti-IL-17 and APC-conjugated anti-interferon gamma (IFNgamma) antibodies using Foxp3 staining buffer kit (eBioscience, San Diego, CA) and analyzed on FACSCalibur (BD Biosciences). IL-17 levels in the culture supernatants were analyzed by enzyme linked immunosorbent assay (ELISA) with a commercial available kit (R&D Systems) according to the manufacture’s instruction.

### Systemic Transplantation of SHED into MRL/lpr Mice

The protocol for systemic transplantation of SHED into MRL/*lpr* mice was referred to our previous reports [9], [13]. Briefly, SHED (0.1×10^6^/10 g body weight) or PBS were systemically infused into MRL/*lpr* mice via the tail vein at the age of 16 weeks old. Their survival was inspected daily until died. Peripheral blood, urine, kidney, axial lymph nodes and long bones were collected from MRL/*lpr* mice at the age of 20 weeks old according to our previous studies [9], [13].

### Assay for Autoantibodies, Immunoglobulins, Biomarkers and Cytokines in Peripheral Blood Serum, Urine and Tissue Samples

Biological factors in mouse biological samples were measured by ELISA with commercial available kits (anti-double strand DNA [dsDNA] IgG and IgM, anti-nuclear antibody [ANA], albumin and complement C3: alpha diagnostic [San Antonio, TX]; IL-17 and nuclear factor kappaB ligand [sRANKL] [R&D Systems]; C-terminal telopeptides of type I collagen [CTX]: Nordic Bioscience [Herlev, Denmark]) according to the manufactures’ instructions. Creatinine (R&D Systems) and urine protein (Bio Rad, Hercules, CA) were also assayed by colorimetry according to the manufactures’ instructions.

### Flow Cytometric Analysis for Mouse Peripheral Blood Th17

To measure Th17 cells in mouse peripheral blood, mouse PBMNCs were incubated with PerCP-conjugated anti-CD4, FITC-conjugated anti-CD8a, followed by the treatment with R-PE-conjugated anti-IL-17 and APC-conjugated anti-IFNgamma antibodies using a Foxp3 staining buffer kit (eBioscience). The stained cells were analyzed on FACSCalibur (BD Bioscience).

### Histopathological Analysis of Mouse Kidney

Kidney samples were fixed with 4% PFA in PBS overnight. After washed with PBS, some samples were processed for paraffin embedding and cut into 6-µm thick paraffin sections. The others were cut into 8-µm thick cryosections. The paraffin sections were treated with H&E, Gomori trichrome or Periodic Acid Schiff (PAS) staining. The cryosections were stained with anti-mouse complement C3 antibody and treated with CF 633-conjugated-secondary antibodies (Biotium). Finally, they were stained with DAPI (Dojindo) and observed under a BIORVO microscope (BZ-9000, Keyence Japan).

### In vivo Tracing Assay of SHED

To trace the trafficking of SHED transfused in MRL/*lpr* mice, SHED were labeled with carboxyfluorescein diacetate succinimidyl ester (CFSE) labeling. Single suspended SHED population (10×10^6^/ml) was incubated with CFSE solution (Invitrogen) for 10 min at 37°C. The labeled cells (1×10^6^) were intravenously injected into the tail vein of MRL/*lpr* mice. Lymph nodes, kidneys and femurs of the mice were harvested either 24 h or 1 week after the infusion. The tissue samples were fixed with 4% PFA in PBS overnight. Only bone samples were treated with 10% EDTA. All of the samples were cut into 6-µm frozen sections, stained with DAPI (Dojindo) and observed under a microscope.

### Bone Phenotype Analysis

The femoral bone samples were fixed with 4% PFA in PBS overnight and analyzed by micro-computed tomography (microCT) with Skyscan 1076 scanner (Skyscan, Kontich, Belgium). Bone mineral density (BMD) and bone structural parameters including bone volume/trabecular volume (BV/TV), trabecular thickness (Tb.Th), trabecular number (Tb.N), trabecular separation (Tb.Sp) and trabecular space (Tb.Spac) were calculated using CT Analyzer software (Skyscan) according to the manufacture’s instruction. After microCT analysis, the bone samples were decalcified with 10% EDTA and cut into 6-µm paraffin sections. The paraffin sections were treated with H&E staining. Some sections were stained with a tartrate-resistant acid phosphate (TRAP) staining solution containing 0.01% naphthol AS-MX phosphate (Sigma), 50 mM tartrate (Sigma) and 0.06% fast red violet LB salt (Sigma) in 0.1 M acetate buffer (pH 5.0) for 10 min. TRAP-positive cells were counted and quantified as previously [19].

### In vitro Osteoclastic and Osteogenic Assays

Bone marrow were flashed out from the bone cavity of femurs and tibias with heat-inactivated 3% FBS (Equitech-Bio) in PBS. For osteoclastic assay, the bone marrow nucleated cells (BMCs) were seeded at 1×10^6^ per well on the 24-well culture plates with 10 ng/ml macrophage colony stimulating factor (M-CSF) (R&D Systems) and 25 ng/ml sRANKL (PeproTech) in alphaMEM for 5–6 days. The medium was changed on Day 3. The cultures were treated with TRAP staining [19] and the TRAP-positive cells with muitinuclei (n>3) were counted [19]. The BMCs (1×10^6^) were also seeded on 35-mm culture dishes and cultured in an osteogenic medium containing 10% FBS (Equitech-Bio), 2 mM L-glutamine (Nacalai Tesque), 100 µM L-ascorbic acid 2-phosphate (WAKO pure chemicals), 2 mM beta-glycerophosphate (Sigma), 10 nM dexamethasone (Sigma), 100 U/ml penicillin and 100 µg/ml streptomycin (Nacalai Tesque) in alphaMEM [20]. Four weeks after the induction, the osteogenic cultures were stained with 1% Alizarin Red (Sigma). The mineralized area was quantified by using NIH Image-J [13].

### Bone Regeneration in Calvarial Bone Defects

The bone defect models in calvarial bone were generated on immunocompromised mice as described before [11]. Briefly, P3 SHED (4×10^6^) were mixed with HA/TCP carriers (40 mg) (Zimmer). Calvarial bones, especially parietal bones, were removed to make a critical defect area. The mixtures of SHED and HA/TCP were placed on the defect area and the calvarial skins were sutured. Twelve weeks after the implantation, the calvariae were fixed with 4% PFA in PBS overnight and imaged by Skyscan 1076 scanner (Skyscan). Then, the samples were decalcified with 10% EDTA and cut into 6-µm thick paraffin sections. The sections were treated with H&E staining and TRAP staining. Some of sections were used for immunofluorescence with anti-human CD146 antibody.

### Statistical Analysis

Student’s *t*-test was used to analyze significance between 2 groups. A *P* value of less than 0.05 was considered as a significant difference.

## Results

### SHED-Cryo Possess MSC Properties

**Figure 1:**
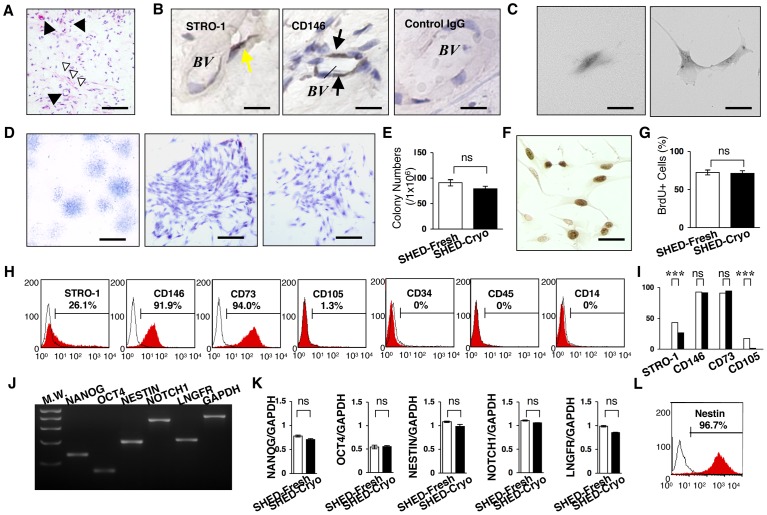
Clonogenicity, cell proliferation capacity and stem cell marker expression of SHED-Cryo. (**A**) Histology of cryopreserved dental pulp tissue of exfoliated deciduous teeth. Black arrowheads: blood vessel, white arrowheads: nerve fibers. H&E staining. (**B**) Localization of MSC markers in the cryopreserved deciduous pulp tissues. Yellow arrows: STRO-1-positive cells, black arrows: CD146-positive cells. *BV*: blood vessel, Control: subclass-matched antibody staining. (**C–E**) CFU-F assay. Formation of a clonogenic cell cluster from a single attached cell (**C**). Images of attached colonies of SHED-Cryo. Toluidine blue staining (**D**). Comparison of CFU-F number (**E**). (**F, G**) Cell proliferation assay. Immunostaining of BrdU-positive nuclei. (**F**). Comparison of cell proliferation (**G**). (**H, I**) Flow cytometry of MSC markers in SHED-Cryo. Representative histograms (**H**). Comparison of STRO-1, CD146, CD73 and CD105. Black columns: SHED-Cryo, white columns: SHED-Fresh (**I**). (**J, K**) Gene expression of embryonic stem and neural crest cell markers. MW: molecular weight markers (**J**). Comparative analysis of *NANOG*, *octamer 4* (*OCT4), NESTIN, NOTCH1* and *low affinity nerve growth factor receptor (LNGFR)* (**K**). (**L**) Flow cytometry of Nestin in SHED-Cryo. **A, B:** n = 3. **C–L:** n = 5 for all group. **A–E:** Bar = 30 µm (**A**), 5 µm (**B, C**, **F**), 1 mm (**D**, left) 25 µm (**D**, middle and right). **G, I, K:** ***P<0.005. ns: no significance. The graph bars represent mean±SD.

SHED have been elucidated to possess MSC characteristics including clonogenicity, self renew, multipotency, *in vivo* regeneration and *in vitro* immunomodulatory functions [10], [13]. Here, we demonstrated the effects of cryopreservation of deciduous pulp tissues on these SHED properties. Dental pulp tissues were removed from exfoliated deciduous teeth and frozen in the freezing medium (10% DMSO and 90% FBS) at −80°C overnight followed by the preservation in a liquid nitrogen-filled tank for over 2 years (**Figure S1**). The remaining tooth bodies were returned to the donor children. The long-term cryopreserved tissues were quickly thawed at 37°C before used (**Figure S1**). Histological analysis confirmed that the cryopreserved tissues showed dense connective tissues containing blood vessels and nerve fibers (**Figure 1A**), but not odontoblastic cells. The odontoblast layer may be lost because of mechanical damage or freezing fracture of the pulp samples. Early MSC markers STRO-1 and CD146-positive cells were detected at the perivascular area (**Figure 1B**) as dental pulp stem cells (DPSCs) were localized around the blood vessels in adult dental pulp tissues [21]. These data suggested a possibility of the remaining of MSCs in the cryopreserved deciduous pulp tissues.

Next, we isolated MSCs from the cryopreserved dental pulp tissues of exfoliated deciduous teeth, SHED-Cryo, by CFU-F approach (**Figure S1**), which is a classical and standard method [15]. When seeded at low density, single cells were adhered to the plastic culture dishes and then divided to generate cell clusters (**Figure 1C**). The clonogenic cell clusters exhibited different size and varied density (**Figure 1D**). The colony forming efficiency of SHED-Cryo showed similar to that of SHED-Fresh (**Figure 1E**). BrdU incorporation assay demonstrated that the proliferation capacity of SHED-Cryo maintained at a high level (**Figure 1F**) similar to that of SHED-Fresh (**Figure 1G**). Flow cytometric analysis verified that SHED-Cryo were positive to STRO-1, CD146, CD73 and CD105 but negative to hematopoietic cell markers CD34, CD45 and CD14 (**Figure 1H**). SHED-Cryo were also positive to CD90 (over 95%) and the positive level in SHED-Cryo was similar to that in SHED-Fresh (data not shown). SHED-Cryo shared the immunophenotype with SHED-Fresh (**Figure 1I**). RT-PCR demonstrated that SHED-Cryo expressed genes for embryonic stem cells, *NANOG* and *octamer 4*, and for neural crest cells, *NOTCH1*, *NESTIN* and *low-affinity neural growth factor* (**Figure 1J**), as seen in SHED-Fresh (**Figure 1K**). Expression of a neural crest cell marker, Nestin, was also detected in SHED-Cryo by flow cytometry (**Figure 1L**) and the expression in SHED-Cryo showed a similar level to that in SHED-Fresh (data not shown). Recent studies [22], [23] demonstrated the effect of cryopreservation on the expression of NANOG, octamer 4 and Nestin in deciduous teeth stem cells and may support, at least in partially, our cryopreserved effect of deciduous dental pulp tissues on SHED properties. Taken together, these data suggested that SHED-Cryo retained primitive MSC properties likely to SHED-Fresh.

### SHED-Cryo Own Multipotency

**Figure 2:**
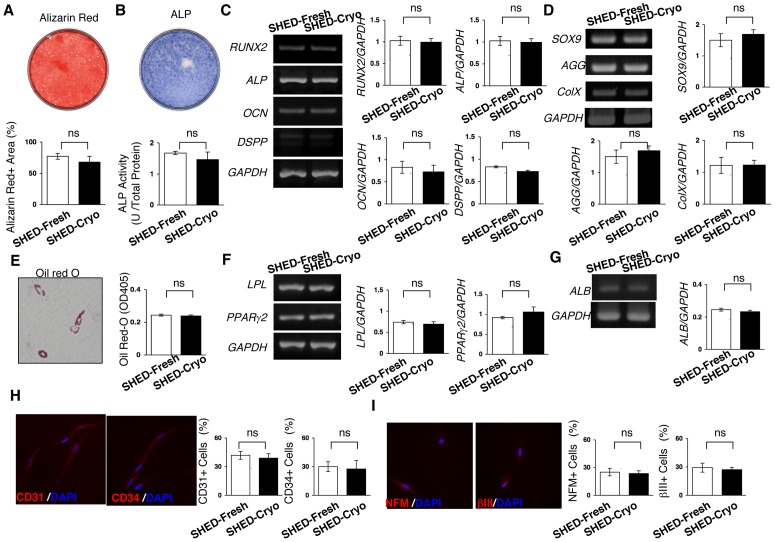
Multipotency of SHED-Cryo. (**A–C**) Dentinogenic/osteogenic differentiation capacity. Images of Alizarin Red staining (**A**) and alkaline phosphatase (ALP) activity (**B**) of SHED-Cryo. Comparison of Alizarin Red-positive (Alizarin Red+) area (**A**), ALP activity (**B**) and odontoblast/osteoblast-specific genes, *runt-related gene 2 (RUNX2), ALP, osteocalcin (OCN), and dentin sialophosphoprotein (DSPP)* (**C**). (**D**) Chondrogenic differentiation capacity. Comparison of chondrocyte-specific genes, *SOX9*, *aggrecan (AGG)* and *type X collagen (ColX)*. (**E, F**) Adipogenic differentiation assay. A representative image of Oil Red-O staining and comparison of Oil Red-O accumulation (**E**). Comparison of adipocyte-specific genes *lipoprotein lipase (LPL)* and *peroxisome proliferator activated receptor-gamma2 (PPARgamma2)* (**F**). (**G**) Hepatogenic differentiation capacity. Comparison of hepatocyte-specific gene *albumin (ALB)*. (**H**) Endothelial cell differentiation assay. Comparison of endothelial cell markers CD31 and CD34. (**H**) Neural cell differentiation assay. Comparison of neural cell markers neurofilament M (NFM) and tubulin betaIII (betaIII). **A–I:** n = 5 for all group. ns: no significance. The graph bars represent mean±SD.

Four weeks after dentinogenic/osteogenic induction, SHED-Cryo were capable of forming Alizarin Red-positive nodules (**Figure 2A**). SHED-Cryo showed a high ALP activity (**Figure 2B**) and expressed odontoblast/osteoblast-specific genes *runt-related gene* 2, *ALP*, *osteocalcin*, and *dentin sialophosphoprotein* (**Figure 2C**) after the 1-week induction. SHED-Cryo expressed chondrocyte-specific genes for *SOX9*, *aggrecan* and *type X collagen* 3 weeks after chondrogenic culture (**Figure 2D**). SHED-Cryo expressed adipocyte-like phenotypes including accumulation of Oil red-O-positive droplets (**Figure 2E**) and expression of adipocyte-specific genes *lipoprotein lipase* and *peroxisome proliferator activated receptor-gamma2* (**Figure 2F**) 6 weeks after adipogenic induction. SHED-Cryo expressed albumin gene, one of hepatocyte-specific genes, 4 weeks after hepatogenic induction (**Figure 2G**). Immunofluorescence revealed that SHED-Cryo expressed endothelial cell markers CD31 and CD34 1 week after endothelial cell-induction (**Figure 2H**) and neural cell markers neurofilament M and tubulin betaIII 3 weeks after neural cell-induction (**Figure 2I**). SHED-Cryo also exhibited similar capabilities of differentiating into odontoblasts/osteoblasts, adipocytes, chondrocytes, hepatocytes, endothelial cells and neural cells to SHED-Fresh (**Figure 2**). These data indicated that SHED-Cryo maintained multipotency as MSCs.

### SHED-Cryo Showed *in vivo* Mineralized Tissue Regeneration

**Figure 3:**
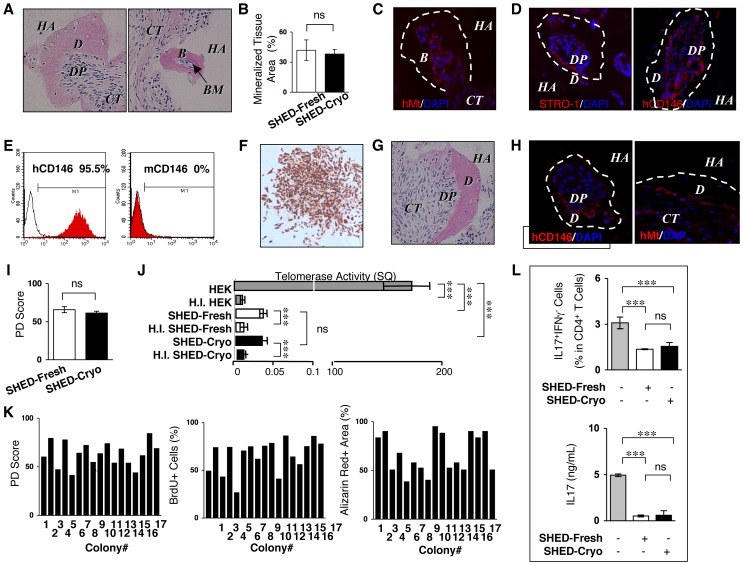
Tissue regeneration capability, self-renewal potency, heterogeneity and *in vitro* immunomodulatory functions of SHED-Cryo. (**A–D**) Images of primary transplant tissues of SHED-Cryo. H&E staining (**A**). Comparison of newly formed-mineralized tissue (B). Immunofluorescence with anti-human specific mitochondria (hMt) (**C**) and anti-STRO-1/human CD146 (hCD146) (**D**) antibodies. (**E, F**) Purity of hCD146 antibody-sorted cells from primary transplants. Flow cytometry with hCD146 and mouse CD146 (mCD146). (**E**). Immunocytochemistry with hCD146 antibody of sorted cell-derived CFU-F (F). (**G, H**) *in vivo* self-renewal assay. Images of secondary transplant tissues. H&E staining (**G**). Immunofluorescence with anti-hCD146/anti-Mt antibodies. (**H**). (**I**) Comparison of population doubling (PD) scores. (**J**) Comparison of telomerase activity. (**K**) Single-colony-derived cell assay with 17 single cell colonies from a cryopreserved deciduous pulp tissues. (**L**) *In vitro* direct immunosuppressive effects of SHED-Cryo on human Th17 cells. **A–J, L**: n = 5 for all group. **A, C, D, F, H**: *B*: bone, *BM*: bone marrow, *CT*: connective tissue, *D*: dentin, *DP*: dental pulp, *HA*: HA/TCP, **I**: HEK: HEK293 cells, H.I. HEK: heat inactivated HEK, H.I. SHED-Cryo: heat inactivated SHED-Cryo, H.I. SHED-Fresh: heat inactivated SHED-Fresh. **C, D, H**: Dot lined areas: mineralized tissue. Nuclei are counterstained with DAPI. **B, H, I, L**: ***P<0.005, ns: no significance. The graph bars represent mean±SD.

Eight weeks after subcutaneous transplantation of SHED-Cryo with HA/TCP carriers into immunocompromised mice (**Figure S2**), dentin/pulp-like complex and bone/bone marrow-like structures were formed around the surface of HA/TCP by histological analysis (**Figure 3A**). Immunofluorescence showed that human mitochondria-positive cells were arranged on mineralized matrix (**Figure 3C**). Human specific STRO-1 and CD146 antibodies-positive cells were also detectable on the regenerated mineralized matrix (**Figure 3D**). On the other hand, control transplant-tissues that implanted only HA/TCP carriers without SHED-Cryo did not express any mineralized tissue and human specific antibody-positive cells (**Figure S3**). Therefore these results suggested that SHED-Cryo were responsible cells for mineralized tissue formation in the implant tissues. SHED-Cryo formed similar amount of the regenerated mineralized tissues when compared to SHED-Fresh (**Figure 3B**). These data indicated that SHED-Cryo retained a unique *in vivo* regenerative capacity likely to SHED-Fresh. Although the origin of bone marrow cells are host cells in bone marrow MSC-implants [24], [25], the origin of cells in bone marrow and dental pulp in SHED-implants have not been elucidated. Furthermore study will be needed to clarify the origin of the dental pulp cells and bone marrow cells in the SHED-implants in future.

### SHED-Cryo Retain Self-renewal Capability

To evaluate self-renewal capability of SHED-Cryo, sequential transplantation, which is one of traditional and gold standard methods to identify the self-renewal capability of stem cells [17], was performed (**Figure S2**). CD146 is considered to be a critical cell surface marker for human MSCs [18]. Cell population was isolated from the primary implants and stained with human CD146 antibody. Then human CD146 antibody-positive cells were purified by a magnet sorting system. Flow cytometric analysis confirmed that the sorted cells were positive to human CD146 (>95%) but negative to mouse CD146 (0%) (**Figure 3E**), evaluating the high purity of the sorted cells as human stem cells. When the sorted cells were seeded at low density, they were capable of forming CFU-Fs that exhibited positive to human CD146 by immunostaining (**Figure 3F**), indicating that the CD146-positive human cells maintained as intact MSCs in the transplant tissues after the long-term implantation. After the colony-forming cells were secondarily transplanted into immunocompromised mice for 8 weeks, the secondary implants contained similar dentin/pulp complex-like structures (**Figure 3G**) to the mineralized structural complexes in the primary transplants. Cells positive to anti-human CD146 or anti-human specific mitochondria antibodies were localized on the mineralized matrix (**Figure 3H**). Population doubling and telomerase activity are associated with self-renewal potential of stem cells [26]. Population doubling assay indicated a prolonged and time-limited cell proliferation in SHED-Cryo (**Figure 3I**). Telomerase activity test revealed the lower activity in SHED-Cryo (**Figure 3J**). Collectively, these results verified that SHED-Cryo represented a self-renewal capacity.

### SHED-Cryo are Heterogeneous Population

To identify the heterogeneity of SHED-Cryo, total 17 clonogenic single-colonies were acquired from a cryopreserved dental pulp tissue of exfoliated deciduous tooth. Each single-colony-derived SHED-Cryo showed various population-doubling score, diverse cell proliferation capacity and varied Alizarin red-positive area (**Figure 3K**). These findings indicated that SHED-Cryo displayed heterogeneous as seen in SHED-Fresh [10].

### Immunomodulatory Properties of SHED-Cryo *in vitro*

To explore whether SHED-Cryo display immunomodulatory capacity to Th17 cells as seen in SHED-Fresh (**Figure 3L**) [13], SHED-Cryo were co-cultured with anti-CD3 and anti-CD28 antibodies-activated human CD4^+^CD25^−^ T cells under the stimulation with IL-6 and TGFbeta_1_. SHED-Cryo were able to inhibit both the differentiation of CD4^+^IL17^+^IFNgamma^−^ Th17 cells and the secretion of IL-17 (**Figure 3L**), suggesting that SHED-Cryo maintained *in vitro* inhibitory effect on Th17 cells.

### SHED-Cryo Transplantation Prolongs the Life Span and Improves Autoimmune Disorder and Renal Dysfunction in MRL/*lpr* Mice

**Figure 4:**
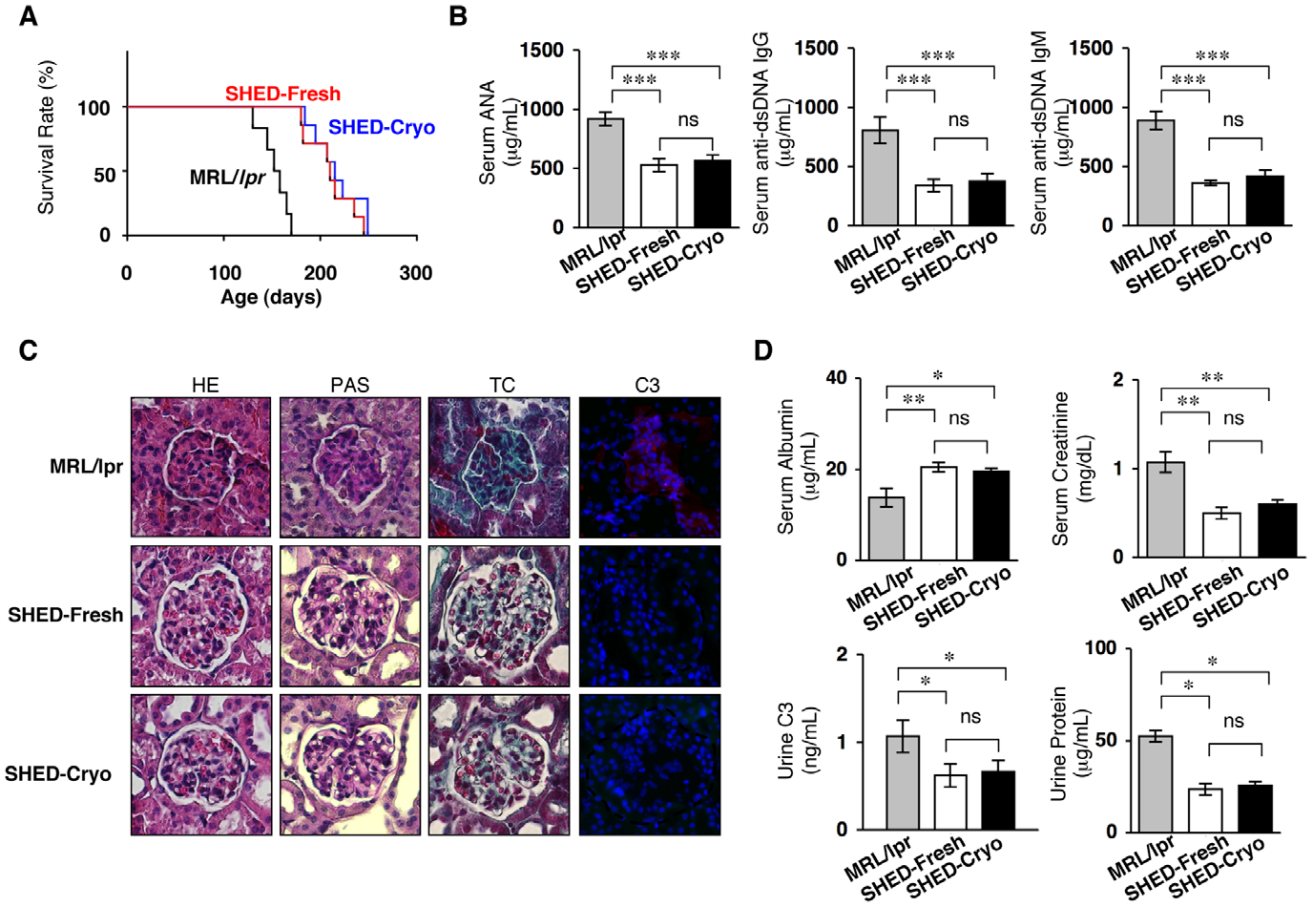
Systemic SHED-Cryo-transplantation improves lifespan and SLE-like disorders in MRL/*lpr* mice. (**A**) Kaplan-Meier survival curve of MRL/*lpr* mice. (**B**) ELISA of serum levels of autoantibodies ANA and anti-dsDNA IgG and IgM antibodies. (**C**) Histopathology of kidneys. *G* and dot-circled area: glomerular. HE: H&E staining, TC: Gomori trichrome staining, PAS: Periodic acid-Schiff staining, C3: Immunofluorescence of Complement C3. DAPI staining. (**D**) Levels of serum albumin and creatinine and urine C3 and protein. **A**–**D**: MRL/*lpr*: control group, SHED-Cryo: SHED-Cryo-transplant group, SHED-Fresh: SHED-Fresh-transplant group. **A**: n = 7, **B**–**D**: n = 5 for all group. **B, D**: *P<0.05, **P<0.01, ***P<0.005, ns: no significance. The graph bars represent mean±SD.

SLE-like autoimmune disorders appear around age 7–8 weeks in MRL/*lpr* mice. Peripheral levels of autoimmune antibodies increase extremely from about 12 weeks of age in MRL/*lpr* mice. To evaluate the therapeutic potency of SHED-Cryo in severed SLE condition as seen in previous studies [13], SHED-Cryo were intravenously transfused to human SLE model MRL/*lpr* mice at the age of 16 weeks old (**Figure S4**). Kaplan-Meyer curve demonstrated that systemic SHED-Cryo-transplantation significantly prolonged the lifespan of MRL/*lpr* mice (**Figure 4A**). Mantel-Haenszel test evaluated that the median survival time was 155.0, 219.0 and 212.5 days in control, SHED-Fresh-transplanted and SHED-Cryo-transplanted MRL/*lpr* mice, respectively. Elevated serum levels of autoantibodies to ANA and anti-dsDNA IgG and IgM antibodies, which are critical clinical markers in human SLE therapy, were markedly decreased in SHED-Cryo transplanted MRL/*lpr* mice **(** **Figure 4B**). Increased peripheral immunoglobulins including IgG_1_, IgG_2a_, IgG_2b_ and IgM were also significantly reduced in SHED-Cryo-transplanted mice (**Figure S5**). Histopathological analysis demonstrated that SHED-Cryo-transplantation prevented renal nephritis associated with hypercellularity, mesangial matrix hyperplasia and basal membrane disorder in MRL/*lpr* mice (**Figure 4C**). Immunofluorescence showed that complement C3-deposition in the glomeruli of the kidney in MRL/*lpr* mice was disappeared after SHED-Cryo-transplantation (**Figure 4C**). SHED-Cryo-transplantation elevated serum albumin level and reduced urine protein (**Figure 4D**), meanwhile, decreased serum creatinine in MRL/*lpr* mice (**Figure 4D**). SHED-Cryo-transplantation displayed similar therapeutic effects on the lifespan, autoantibody levels and renal function in MRL/*lpr* mice with SHED-Fresh-transplantation (**Figures 4** **and S5**). These findings provided that SHED-Cryo retained therapeutic efficacy on MRL/*lpr* mice.

### Transplantation of SHED-Cryo Suppresses Peripheral Th17 Cells in MRL/*lpr* Mice

**Figure 5:**
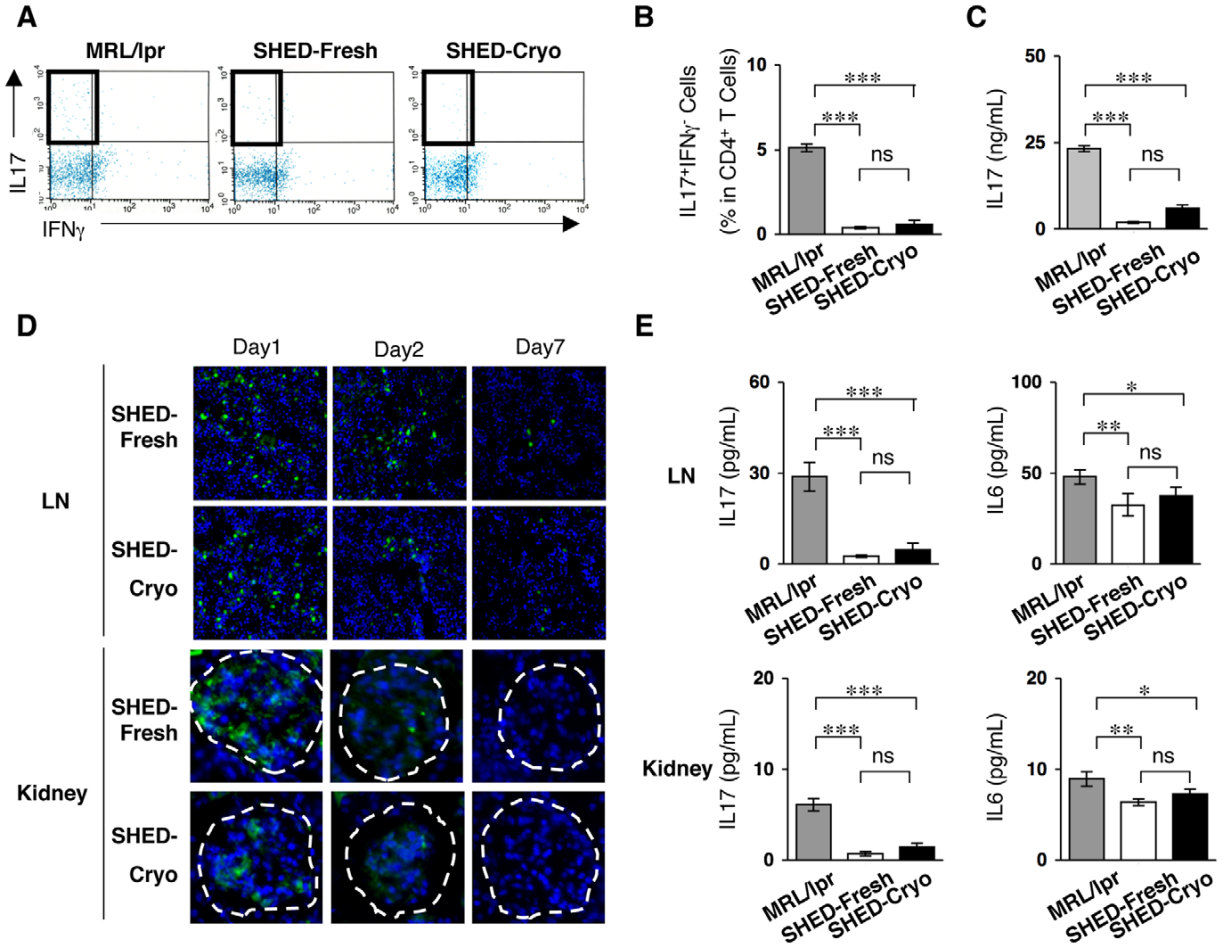
SHED transplantation suppresses circulating and local levels of Th17 cells in MRL/*lpr* mice. (**A, B**) Flow cytometry of peripheral CD4^+^IL17^+^IFNgamma^−^ Th17 cells. (**C**) Serum levels of IL-17. (**D**) Homing of systemically infused CFSE-labeled SHED-Cryo and SHED-Fresh to lymph node (LN) and kidney of MRL/*lpr* Mice after 1- (Day 1), 2- (Day 2) or 7- (Day 7) day transplantation. Dot-circled area: glomerular. (**E**) ELISA of IL-17 an IL-6 levels in lymph node and kidney. **A**–**E**: n = 5 for all group. MRL/*lpr*: control group, SHED-Cryo: SHED-Cryo-transplant group, SHED-Fresh: SHED-Fresh-transplant group. **B, C, E**: *P<0.05, **P<0.01, ***P<0.005, ns: no significance. The graph bars represent mean±SD.

Th17-cell regulation is a critical therapeutic strategy for SLE treatment [27]. The present study showed that the levels of peripheral Th17 cells and IL-17 were remarkably reduced in SHED-Cryo-received MRL/*lpr* mice at the age of 20 weeks old (**Figures 5A–5C**). This Th17-cell suppressive effect was similar to the effect of SHED-Fresh-transplantation on MRL/*lpr* mice (**Figures 5B and 5C**) [13], suggesting that SHED-Cryo maintained *in vivo* immunomodulatory functions.

### Systemically Infused SHED-Cryo Home to Lymph Node and Kidney in MRL/*lpr* Mice and Regulate the Local Immune Microenvironment

To assess the homing ability of SHED-Cryo into sites of injured tissues, CFSE-labeled SHED-Cryo were intravenously injected into MRL/*lpr* mice at the age of 16 weeks old. The high frequency of CFSE-positive SHED-Cryo was observed in the lymph nodes and kidneys, particularly in the glomeruli, on one day after the transplantation (**Figures 5D and S** **6**). The frequency of CFSE-positive SHED-Cryo decreased gradually in both tissues from the 2nd day to 7th day after the transplantation (**Figures 5D and S** **6**). The localization of SHED-Cryo were similar to that of SHED-Fresh (**Figures 5D and S** **6**). The levels of inflammatory cytokines IL-17 and IL-6 in the lymph nodes and kidneys were significantly reduced in SHED-Cryo-transfused MRL/*lpr* mice, as well as SHED-Fresh-transfused mice, when compared to control MRL/*lpr* mice **(** **Figure 5E** **)**. Taken together, these *in vivo* studies suggested that SHED-Cryo were capable of homing to lesional sites and might improve the pathological environments of damaged tissues.

### Systemic SHED-Cryo-transplantation Improves Osteoporotic Skeletal Disorder in MRL/*lpr* Mice

**Figure 6:**
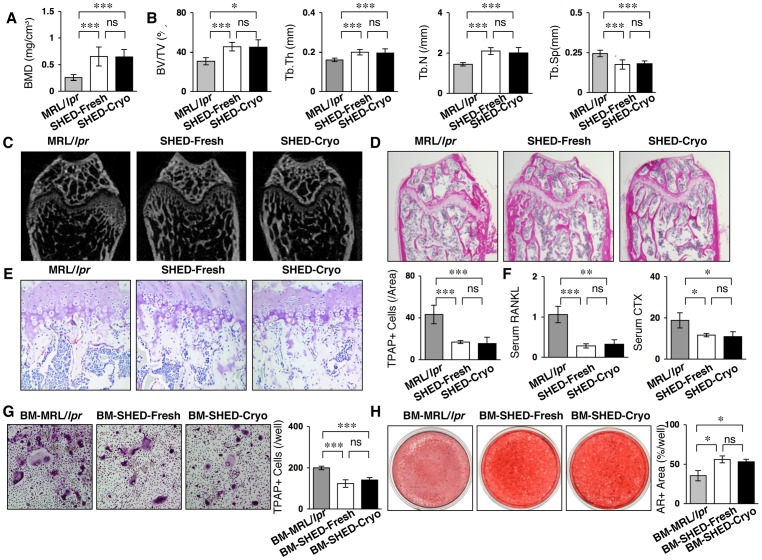
SHED-Cryo transplantation ameliorates osteoporotic bone disorder in MRL/*lpr* mice. (A, B) MicroCT analysis of tibiae. BMD (**A**). Trabecular parameters, bone volume ratio to tissue volume (BV/TV), trabecular thickness (Tb.Th), and trabecular number (Tb.N) along with increased trabecular separation (Tb.Sp) (**B**). (**C, D**) MicroCT (**C**) and histological (**D**) images of trabecular bone structures of tibiae. H&E staining (**D**). (**E**, **F**) *In vivo* osteoclast activity. TRAP staining (**E**). ELISA of serum sRANKL and C-terminal telopeptides of type I collagen (CTX) (**F**). (**G**) *Ex vivo* sRANKL-induced osteoclastogenesis. TRAP+ cells: TRAP-positive osteoclast-like cells. (**H**) *Ex vivo* osteogenic capacity. Alizarin red-positive (AR+) area after four-week induction. **A–H:** n = 5 for all groups. **A–F:** MRL/*lpr*: control group, SHED-Cryo: SHED-Cryo-transplant group, SHED-Fresh: SHED-Fresh-transplant group, **G, H:** BM-MRL/*lpr*: control MRL/*lpr* mice-derived bone marrow cells, BM-SHED-Cryo: SHED-Cryo-transplanted mice-derived bone marrow cells, BM-SHED-Fresh: SHED-Fresh-transplanted mice-derived bone marrow cells. **A, B, E, F:** *P<0.05, ***P<0.005 (vs. MRL/*lpr*). **G, H:** *P<0.05, **P<0.01, ***P<0.005 (vs. BM-MRL/*lpr*), ns: no significance. **A, B, E–H:** The graph bars represent mean±SD.

The homing ability of SHED-Cryo into bones was analyzed 1 day after the infusion into MRL/*lpr* mice at the age of 16 weeks old. The CFSE-positive SHED-Cryo was sparsely observed in the bone marrow space of MRL/*lpr* mice 7 days after the infusion (**Figure S7**). The frequency of CFSE-positive SHED-Cryo was similar to that of SHED-Fresh (**Figure S7**). MRL/*lpr* mice expressed a remarkable osteoporotic bone-loss in their long bones (**Figures 6A–6D**). Systemic SHED-Cryo-transplantation was capable of increasing BMD and recovering trabecular bone structures in the long bones of MRL/*lpr* mice (**Figures 6A and 6B**). TRAP-positive cells were significantly reduced in the long bones of SHED-Cryo-transplanted group compared to the non-transplanted control group (**Figure 6E**). SHED-Cryo-transplantation markedly reduced serum levels of sRANKL and CTX in MRL/*lpr* mice (**Figure 6F**). Flow cytometry revealed that CD4^+^IL-17^+^IFNgamma^−^ Th17 cells were significantly reduced in bone marrow cells of SHED-Cryo- and SHED-Fresh-transplanted MRL/*lpr* mice compared to the control MRL/*lpr* mice (**data not shown**), suggesting that immunomodulatory functions of SHED-Cryo and SHED-Fresh may contribute to reduce the bone reduction in MRL/*lpr* mice. To examine *ex vivo* osteoclastogenesis and osteogenesis, BMCs were isolated from control, SHED-Fresh-transplanted and SHED-Cryo-transplanted MRL/*lpr* mice, termed control-, SHED-Fresh- and SHED-Cryo-BMCs, respectively. When BMCs were stimulated with M-CSF and sRANKL, the number of TRAP-positive multinucleated cells was significantly reduced in SHED-Cryo-BMCs than in control-BMCs (**Figure 6G**). Osteogenic analysis showed that Alizarin Red-positive area was shared larger in SHED-Cryo-BMCs than control-BMCs (**Figure 6H**). SHED-Cryo-transplanted MRL/*lpr* mice expressed similar bone regenerative effects to SHED-Fresh-transplanted MRL/*lpr* mice (**Figure 6**). These data indicated that SHED-Cryo-transplantation improved osteoporotic disorder in MRL/*lpr* mice. Furthermore studies will be needed to evaluate the therapeutic mechanism of SHED to osteoporotic disorder in immune diseases likely SLE.

### SHED-Cryo-implantation Repairs the Calvarial Bone Defects in Immunocompromised Mice

**Figure 7:**
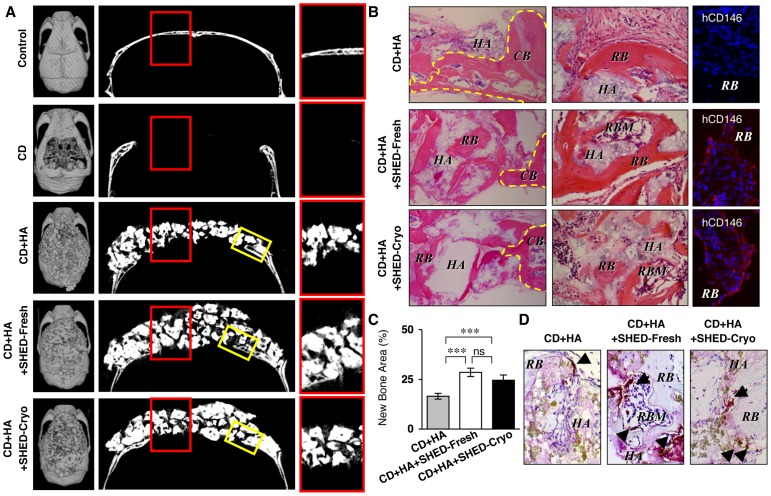
SHED-Cryo are capable of repairing critical calvarial bone defects in immunocompromised mice. (**A**) MicroCT images of mouse calvariae. Left panels: cranial images, middle panels: saggital images, right panels: images of red-bowed area in riddle panels. (**B**) Histology of bone regeneration in mouse calvariae. Left panels: edge parts of the defect area (yellow-boxed area in Figure 7A). H&E staining; middle panels: middle parts of the defect area (red red-bowed area in Figure 7A). H&E staining; right panels: immunofluorescence with anti-human CD146 antibody (hCD146). DAPI staining. CB, yellow dot-circled area: calvarial bone, HA: HA/TCP, RB: regenerated bone, RBM: regenerated bone marrow. (**C**) Regenerated bone area in the defect area. (**D**) Distribution of osteoclasts. Arrowheads: TRAP-positive cells. TRAP staining. **A–D:** n = 5 for all groups. Control: control (non-defect) group, CD: calvarial defect group, CD+HA: HA/TCP-implanted group, CD+HA+SHED-Fresh: SHED-Fresh-implanted group, CD+HA+SHED-Cryo: SHED-Cryo-implanted group. **C:** ***P<0.005, ns: no significance. The graph bars represent mean±SD.

From the present *in vivo* tissue regeneration capability of SHED-Cryo, we hypothesized that SHED-Cryo could regenerate bone tissues in bone defects, as seen in SHED-Fresh (**Figure 7A–C**) [11]. We generated critical calvarial bone defects on immunocompromised mice and implanted SHED-Cryo with HA/TCP carrier onto the defect area (**Figure S8**). SHED-Cryo-implantation was able to regenerate the calvarial defects with a large amount of bone-like structures and bone-marrow-like components compared to implantation with only HA-TCP (**Figure 7A–C**). The amount of regenerated bone and bone marrow at SHED-Cryo-implanted sites was similar to that at SHED-Fresh implanted sites (**Figure 7A–C**). Immunofluorescence with anti-human CD146 antibody revealed that SHED-Cryo were responsible cells for bone regeneration in SHED-Cryo- and SHED-Fresh-implanted group, but not in HA/TCP-implanted group (**Figure 7B**). These findings suggested that SHED-Cryo and SHED-Fresh could be differentiated into bone-forming cells to contribute to repair the bone defect. A large number of TRAP-positive osteoclast-like cells were found in the regenerated bone tissues in SHED-Cryo-implanted sites, as well as in that of SHED-Fresh-implanted sites, but less in HA/TCP-implanted sites (**Figure 7D**), suggesting that the regenerated bone tissues might indicate a physiological bone-remodeling ability by osteoclasts and osteoblasts. These data implied that SHED-Cryo were a useful cell source for tissue engineered bone regeneration. Implanted MSCs are impaired by host lymphocytes through secreting the pro-inflammatory cytokines IFNgamma and TNFalpha to suppress MSC-mediated bone regeneration [28]. On the other hand, deciduous teeth stem cells are capable of regenerating bone tissues in the calvarial defects of non-immunosuppressed rats [29]. Furthermore study will be necessary to clarify the kinetics of SHED under immunocompetent conditions for future clinical tissue engineering.

## Discussion

Since SHED has been identified in dental pulp of deciduous tooth and characterized as MSCs [10], deciduous dental pulp tissues have been considered a promising stem cell source. SHED or deciduous teeth stem cells express multipotency into several lineage cells including of dentin/bone-forming cells [10], [30], endothelial cells [30], neural cells [10], [31] and myocytes [22] *in vitro* and *in vivo*. SHED or deciduous teeth stem cells were also applied for tissue-engineering in large animal models including bone defects, muscular dystrophy and dentin defects [32]–[34], as well as small animal models including bone defect and spinal cord injury [11], [29], [35]. Recent study demonstrates *in vitro* immunomodulatory functions of SHED and evaluates the immune therapeutic efficacy on SLE-like model mice [13]. Herein, we demonstrated the feasibility of cryopreserved dental pulp tissue of human deciduous teeth in SHED-based bone tissue engineering and immune therapy. Our results indicate that long-term cryopreservation of human dental pulp tissues of deciduous teeth provides a great potential in future translational researches and clinical applications.

Postnatal stem cells have offered great promise to care diverse diseases. Besides HSCs have acquired outstanding and extensive success in a variety of human diseases such as leukemia, aplastic anemia and autoimmune diseases over half century [36], [37]. To date, MSCs also admit to regenerative medicine for GVHD [6], skeletal reconstruction [2], [3] and SLE [9]. Several challenges have remained to concern quality and safety in respective processes of MSC transplantation such as the cell processing and preserving *ex vivo*. Traditional bone marrow MSCs significantly reduced their frequency and multipotency donor-age-dependently [38]–[41]. Aspiration of bone marrow might accompany sever invasion to the donors [42]. As these significant disadvantages promote to seek alternative resources with accessible and least invasive approaches, novel MSC populations have been identified from various sources such as adipose tissue, cord blood and dental pulp [43]–[45]. While the isolation process of MSCs is intricate, clinically applicable processing for storing and banking of the resources can offer great advantages for MSC-based therapy as well as the reduction of the number of staffing required for cell processing. Cryopreservation of stem cells has provided several utilities such as long-term storage, adjusting a therapeutic cell dose, reducing contamination for safety and quality in the clinical applications [34], [46]. This approach has been used widely and successfully in bone marrow transplantation [47] and HSC transplantation [48]. Therefore, cryopreserved store and banking of MSC resources would be a variable, indispensable and practical approach for stem cell-based therapy.

SHED have been considered to be a primary promising source for regenerative medicine [10]. Exfoliated deciduous teeth represent the most easily, least invasively accessible and feasible resource because of their natural fate (exfoliation) and clinical abolition. SHED offered profound therapeutic efficacy to skeletal defects [11], [12] and autoimmune disease [13]. On the other hand, several tasks have remained to be solved in SHED processing and banking. It is generally hard to expect a chance of the exfoliating of deciduous teeth and to maintain the tissue activity of the interests for a while after the harvesting. In addition, isolation process of SHED accompanies with several complicated, arduous and time-consuming steps and attentive operations. Recent discoveries about functional MSCs recovered from cryopreserved intact dental pulp and periodontal ligament (PDL) tissues of adult human third molars suggest that the least minimal processing may be adequate for the banking of samples [49], [50]. Recovered MSCs after the cryopreservation can still maintain the immunomodulatory capacity *in vitro* [51], [52]. Deciduous teeth stem cells is known to maintain the stem cell property and multipotency after the long-term cryopreservation [22], [23]. In the present study, we firstly demonstrate that stem cells are capable of recovering from human dental pulp tissues of exfoliated deciduous teeth after long-term (over 2 years) cryopreservation. The recovered SHED retained superior MSC properties such as self-renew, multipotency, *in vivo* dentin/bone-regeneration and *in vitro* immunomodulatory function. In addition, transplantation of SHED-Cryo showed critical therapeutic efficiency on both immune and skeletal disorders in MRL/*lpr* mice and bone defects in the calvariae of immunocompromised mice. Taken together, these data suggest that long-term cryopreservation of dental pulp tissues of deciduous teeth is an innocuous and designable approach for clinical banking of stem cells and gives great advantages in immune therapy and bone tissue engineering of regenerative medicine. Furthermore, the present pulp tissue banking system could allow children and their parents to return the “baby teeth” bodies as the most precious souvenirs to the children’s growing.

Current studies indicate that the biological recovery of the PDL stem cells and adult DPSCs are inferior to the freshly isolated stem cells after the cryopreservation of PDL tissues [50] and adult teeth [49]. Whereas, cryopreserved stem cells from apical papilla (SCAP) show a similar biological and immunomodulatory functions to freshly isolated SCAP [51]. Apical papillae have a responsibility to form and extend tooth roots at the developing stage [53], [54], indicating that apical papillae are an active tissue biologically. Cryopreserved deciduous teeth stem cells can also maintain the stem cell property and multipotency [22], [23]. The present study demonstrated that cryopreservation of deciduous dental pulp did not affect the biological and immunological properties of SHED. Moreover, SHED show higher cell proliferation capability than adult DPSCs [10], suggesting that deciduous pulp tissues maintain greater biological activity than adult dental pulp tissues. The discrepancy of the functionally recovery efficiency among deciduous dental pulps, adult PDLs and adult dental pulps after the cryopreservation might depend on the age and/or potential activity of donor samples, supporting the feasibility of the cryopreservation of deciduous dental pulp tissues.

In conclusion, the present cryopreservation of dental pulp tissues of human exfoliated deciduous teeth does not affect on the biological, immunological and therapeutic functions of SHED. Therefore, cryopreserved approach of deciduous dental pulp tissues not only serve as a most clinically desirable banking approach, but also provide sufficient number of SHED for critical therapeutic benefits to stem cell-based immune therapy and tissue engineering in regenerative medicine.

## Supporting Information

Figure S1 **A scheme of the cryopreservation and isolation of mesenchymal stem cells (MSCs) from dental pulp tissues of exfoliated deciduous teeth.** Deciduous dental pulp tissues in the remnant crown (yellow-dot circled region) were removed *en bloc* mechanically, stored in a freezing medium and preserved in a liquid nitrogen tank over 2 years. The frozen tissues were thawed at 37°C and treated with an enzyme solution. SHED from the cryopreserved deciduous dental pulp tissues (SHED-Cryo), as well as SHED from fresh deciduous dental pulp tissues (SHED-Fresh), were obtained by colony forming units fibroblasts (CFU-F) method.
(PDF)

Figure S2 **A scheme of** ***in vivo*** **tissue regeneration and self-renewal assays of SHED-Cryo.** SHED-Cryo were subcutaneously transplanted with HA/TCP carrier into immunocompromised mice. Eight weeks after the implantation, the primary transplants were harvested. Some transplants were used for histological and immunofluorescent analyses. Cells were isolated from the other primary transplants and stained with human-specific CD146 antibody. Human CD146-positive cells were magnetically sorted. The purity of the cells was confirmed by flow cytometry as described in Materials and Methods. The CD146-positive cells were seeded at low density to obtain CFU-F-forming cells. The colony-forming cells were expanded and transplanted secondarily into immunocompromised mice. The secondary transplants were harvested 8 weeks after the implantation and analyzed morphologically.
(PDF)

Figure S3 **Images of primary transplant tissues with HA/TCP alone.** (**A**) H&E staining (HE). (**B-D**) Immunofluorescence with anti-human specific mitochondria (hMt) (**B**), anti-STRO-1 (**C**) and human CD146 (hCD146) (**D**) antibodies.
(PDF)

Figure S4 **A scheme of the transplantation of SHED-Cryo into MRL/** ***lpr*** **mice (** ***lpr*** **).** SHED-Cryo were infused into MRL/*lpr* mice via the tail vein at the age of 16 weeks. The mice were maintained until died for the survival assay. At 20-week-old, some mice were harvested and biological samples were collected to assess the therapeutic efficacy.
(PDF)

Figure S5 **Systemic SHED-Cryo-transplantation improves levels of serum immunoglobulins in MRL/** ***lpr*** **mice.** n = 5 for all group. *P<0.05, **P<0.01, ***P<0.005, ns: no significance. The graph bars represent mean±SD. MRL/lpr: non-transplanted group, SHED-Fresh: SHED-Fresh-transplanted group, SHED-Cryo: SHED-Cryo-transplanted group.
(PDF)

Figure S6 **Homing of systemically infused SHED-Cryo to lymph node and kidney of MRL/** ***lpr*** **Mice.** Images of CFSE-labeled SHED-Cryo and SHED-Fresh in lymph nodes (LN) and kidneys of MRL/*lpr* mice 1 (Day 1) or 7 (Day 7) days after the transplantation. CFSE: CSFE image, DAPI: DAPI image, SHED-Fresh: SHED-Fresh-infused group, SHED-Cryo: SHED-Cryo-infused group.
(PDF)

Figure S7 **Homing of systemically infused SHED-Cryo to bone of MRL/** ***lpr*** **Mice.** Images of CFSE-labeled cells in bone of MRL/*lpr* mice 7 days after the transplantation. CFSE: CFSE image, DAPI: DAPI image, CFSE/DAPI: Merged image of CFSE and DAPI images, SHED-Fresh: SHED-Fresh-infused group, SHED-Cryo: SHED-Cryo-infused group.
(PDF)

Figure S8 **A scheme of the transplantation of SHED-Cryo into calvarial bone defect of immunocompromised mice.** SHED-Cryo were expanded and mixed with HA/TCP carriers. Calvarial bones, especially parietal bone area (P), were removed to generate a bone defect on immunocompromised mice. SHED & HA/TCP mixture were implanted to cover over the defect area. Twelve weeks after the implantation, the samples were harvested and analyzed by microCT and histology.
(PDF)

Table S1 **The list of antibodies.**
(PDF)

Table S2 **The list of primer pairs for RT-PCR.**
(PDF)
