# Supplementary figures and images for: Cryopreserved Dental Pulp Tissues of Exfoliated Deciduous Teeth Is a Feasible Stem Cell Resource for Regenerative Medicine
Source: PLoS One. 2012 Dec 14;7(12):e51777. doi: 10.1371/journal.pone.0051777 (PMC3522596; doi:10.1371/journal.pone.0051777)

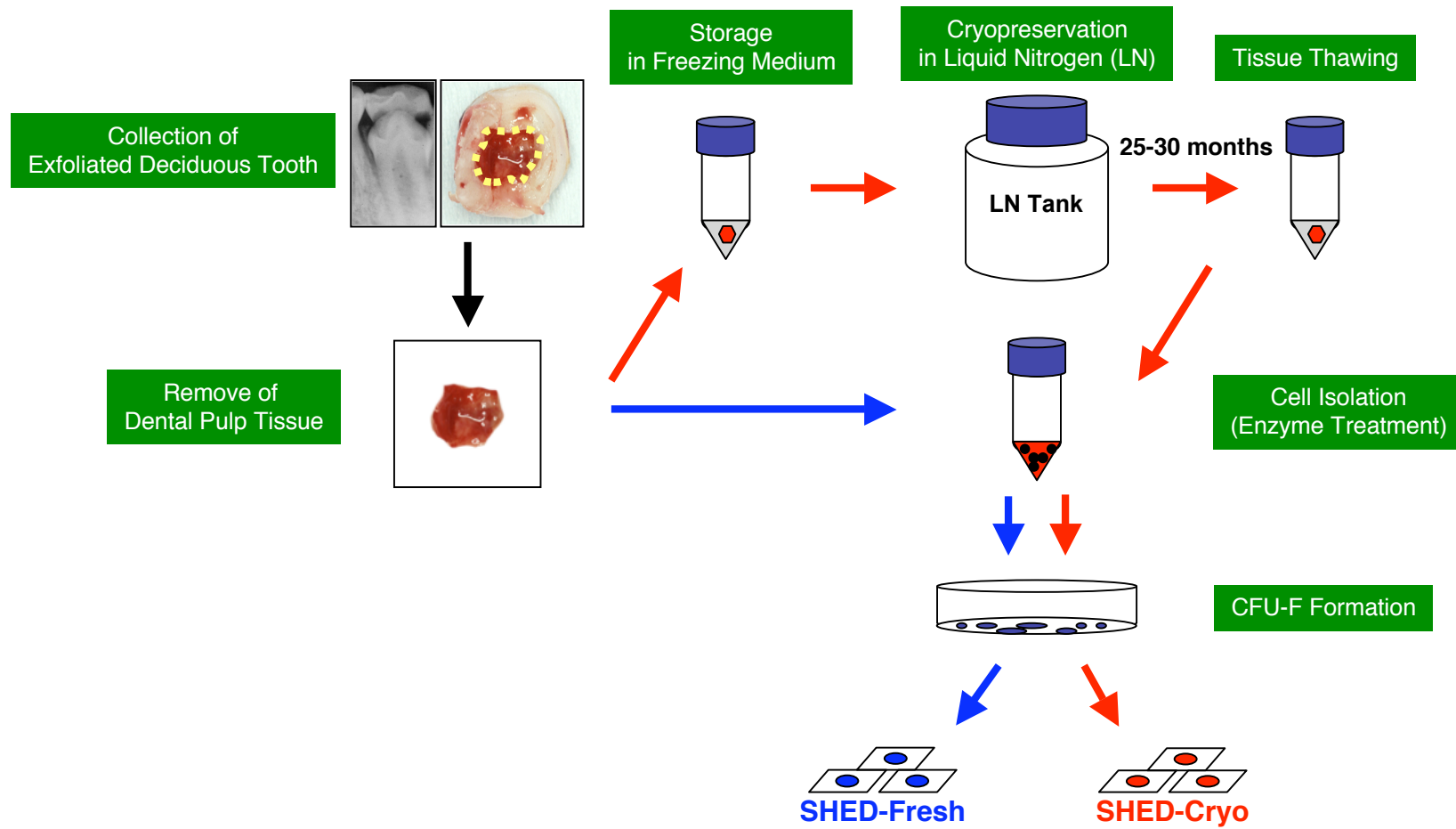

Figure S1

Supplement: Figure S1 — A scheme of the cryopreservation and isolation of mesenchymal stem cells (MSCs) from dental pulp tissues of exfoliated deciduous teeth. Deciduous dental pulp tissues in the remnant crown (yellow-dot circled region) were removed en bloc mechanically, stored in a freezing medium and preserved in a liquid nitrogen tank over 2 years. The frozen tissues were thawed at 37°C and treated with an enzyme solution. SHED from the cryopreserved deciduous dental pulp tissues (SHED-Cryo), as well as SHED from fresh deciduous dental pulp tissues (SHED-Fresh), were obtained by colony forming units fibroblasts (CFU-F) method. (PDF) [file pone.0051777.s001.pdf]

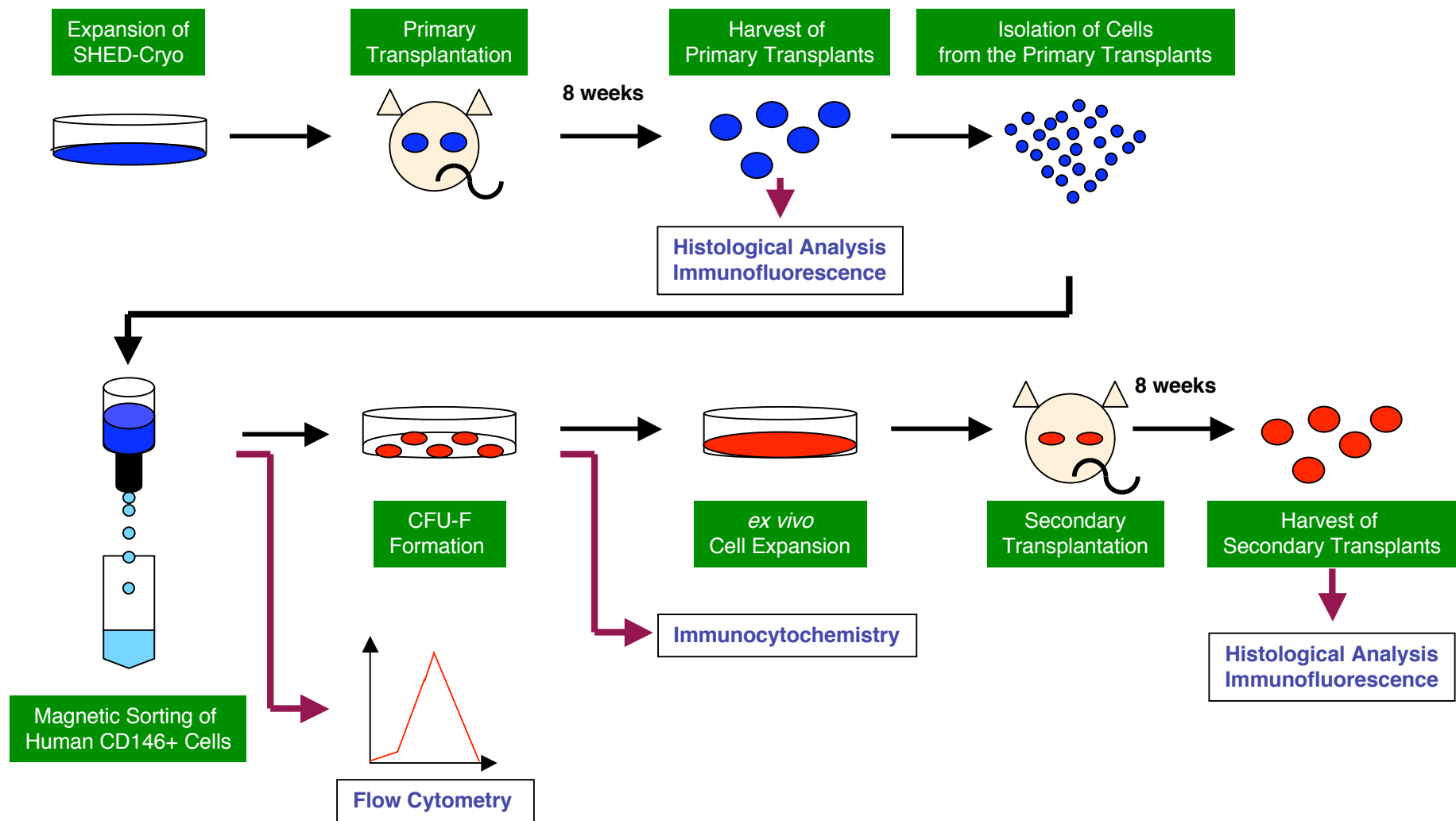

Figure S2

Supplement: Figure S2 — A scheme of in vivo tissue regeneration and self-renewal assays of SHED-Cryo. SHED-Cryo were subcutaneously transplanted with HA/TCP carrier into immunocompromised mice. Eight weeks after the implantation, the primary transplants were harvested. Some transplants were used for histological and immunofluorescent analyses. Cells were isolated from the other primary transplants and stained with human-specific CD146 antibody. Human CD146-positive cells were magnetically sorted. The purity of the cells was confirmed by flow cytometry as described in Materials and Methods. The CD146-positive cells were seeded at low density to obtain CFU-F-forming cells. The colony-forming cells were expanded and transplanted secondarily into immunocompromised mice. The secondary transplants were harvested 8 weeks after the implantation and analyzed morphologically. (PDF) [file pone.0051777.s002.pdf]

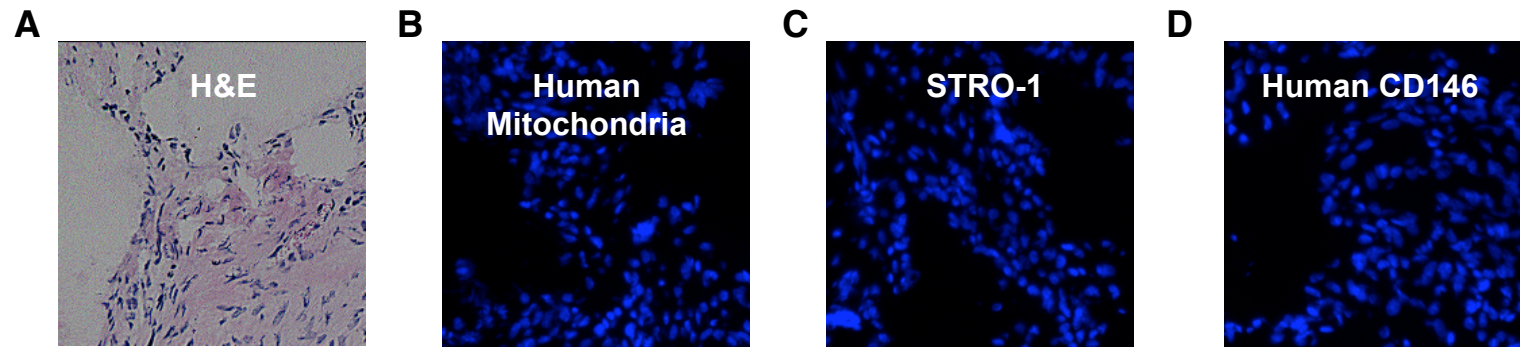

**Figure S3**

Supplement: Figure S3 — Images of primary transplant tissues with HA/TCP alone. (A) H&E staining (HE). (B-D) Immunofluorescence with anti-human specific mitochondria (hMt) (B), anti-STRO-1 (C) and human CD146 (hCD146) (D) antibodies. (PDF) [file pone.0051777.s003.pdf]

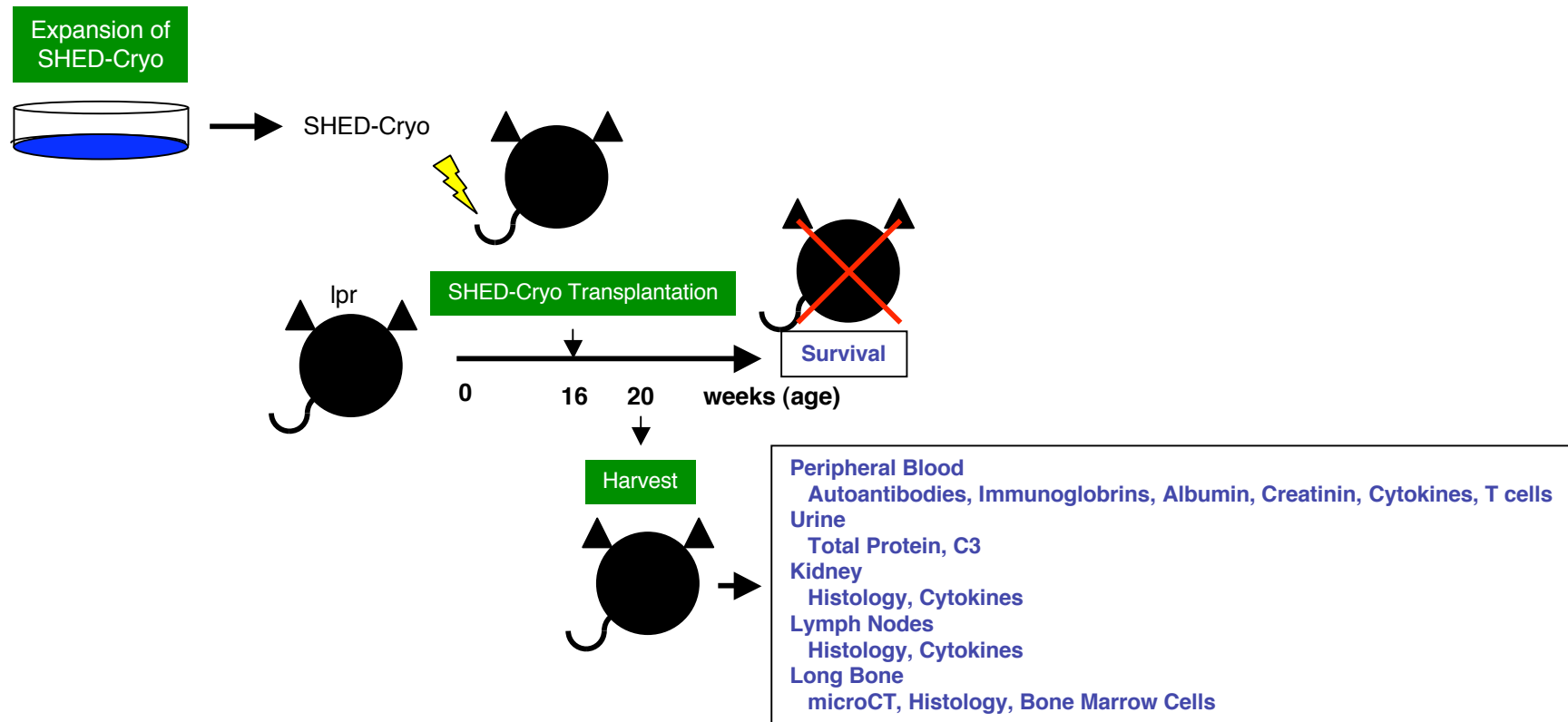

Figure S4

Supplement: Figure S4 — A scheme of the transplantation of SHED-Cryo into MRL/ lpr mice ( lpr ). SHED-Cryo were infused into MRL/lpr mice via the tail vein at the age of 16 weeks. The mice were maintained until died for the survival assay. At 20-week-old, some mice were harvested and biological samples were collected to assess the therapeutic efficacy. (PDF) [file pone.0051777.s004.pdf]

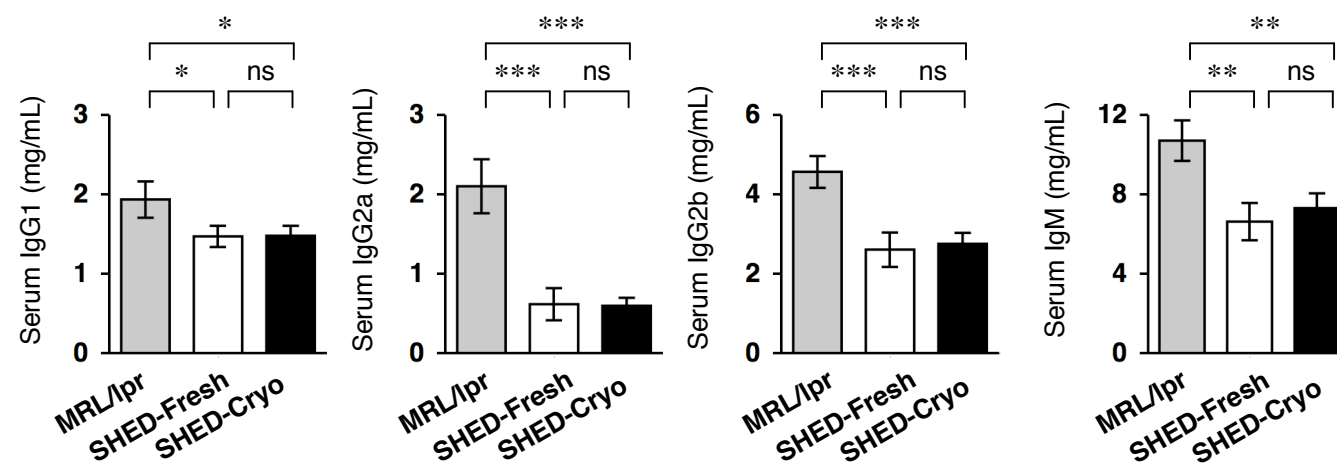

Figure S5

Supplement: Figure S5 — Systemic SHED-Cryo-transplantation improves levels of serum immunoglobulins in MRL/ lpr mice. n = 5 for all group. *P<0.05, **P<0.01, ***P<0.005, ns: no significance. The graph bars represent mean±SD. MRL/lpr: non-transplanted group, SHED-Fresh: SHED-Fresh-transplanted group, SHED-Cryo: SHED-Cryo-transplanted group. (PDF) [file pone.0051777.s005.pdf]

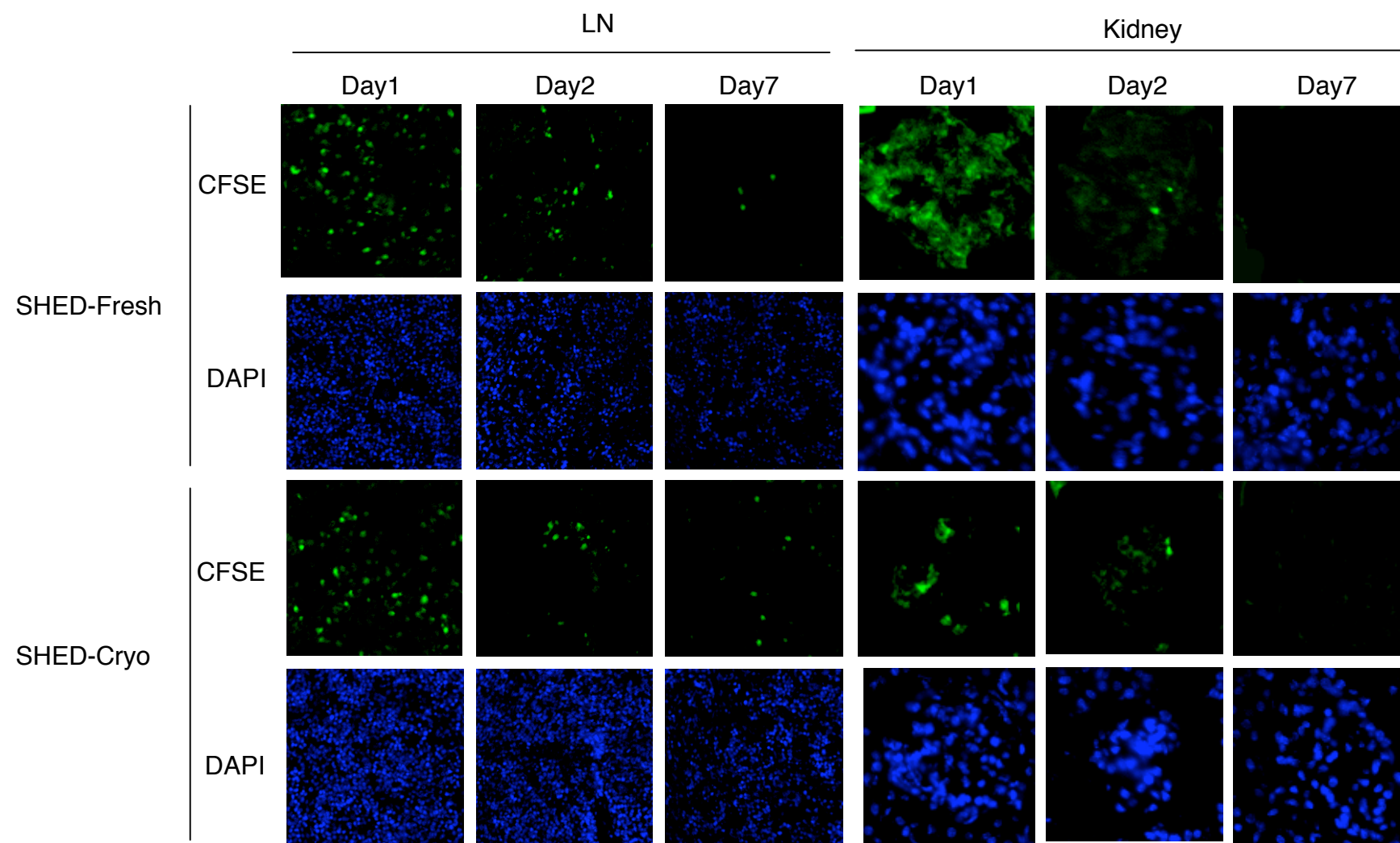

Figure S6

Supplement: Figure S6 — Homing of systemically infused SHED-Cryo to lymph node and kidney of MRL/ lpr Mice. Images of CFSE-labeled SHED-Cryo and SHED-Fresh in lymph nodes (LN) and kidneys of MRL/lpr mice 1 (Day 1) or 7 (Day 7) days after the transplantation. CFSE: CSFE image, DAPI: DAPI image, SHED-Fresh: SHED-Fresh-infused group, SHED-Cryo: SHED-Cryo-infused group. (PDF) [file pone.0051777.s006.pdf]

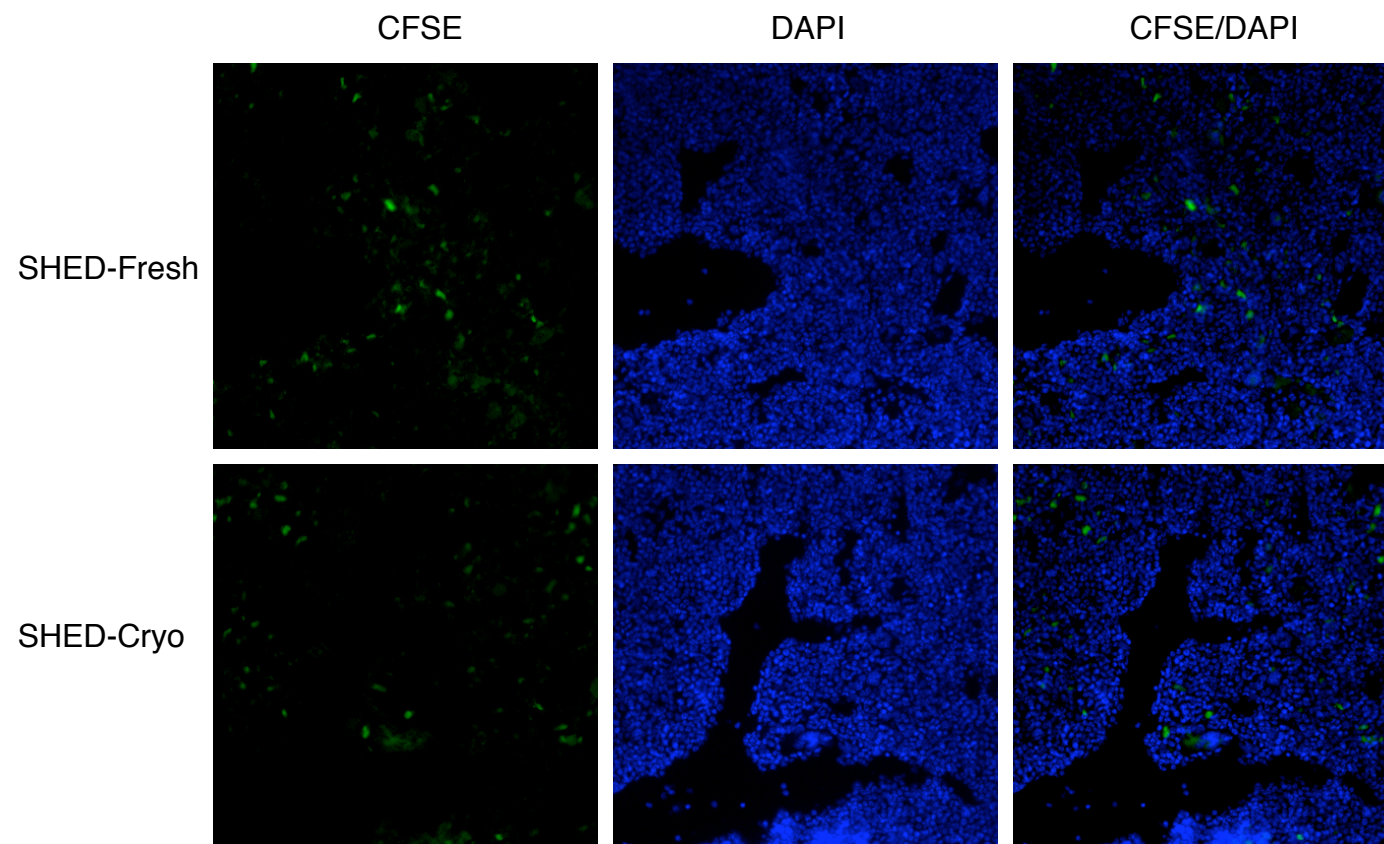

**Figure S7**

Supplement: Figure S7 — Homing of systemically infused SHED-Cryo to bone of MRL/ lpr Mice. Images of CFSE-labeled cells in bone of MRL/lpr mice 7 days after the transplantation. CFSE: CFSE image, DAPI: DAPI image, CFSE/DAPI: Merged image of CFSE and DAPI images, SHED-Fresh: SHED-Fresh-infused group, SHED-Cryo: SHED-Cryo-infused group. (PDF) [file pone.0051777.s007.pdf]

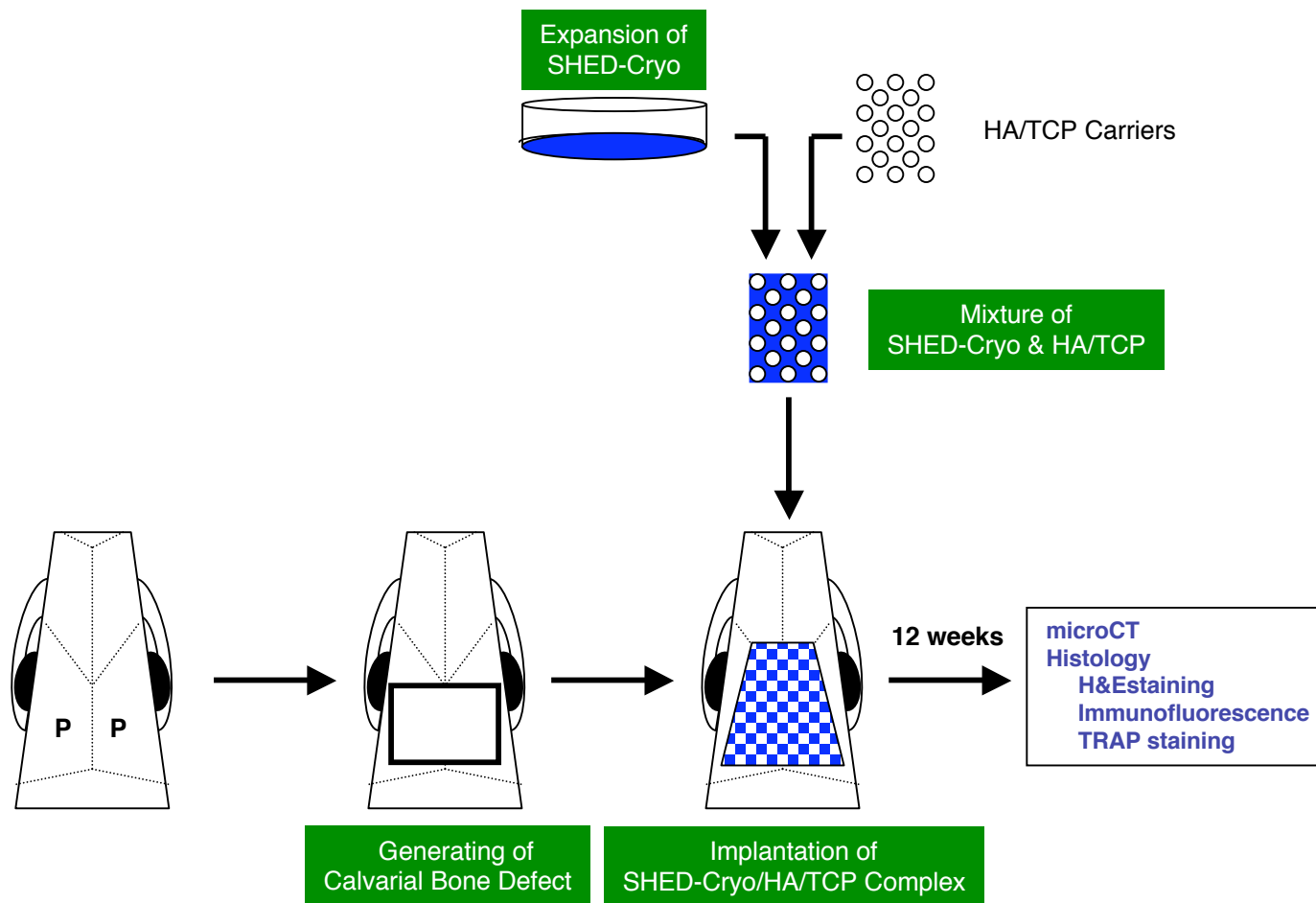

Figure S8

Supplement: Figure S8 — A scheme of the transplantation of SHED-Cryo into calvarial bone defect of immunocompromised mice. SHED-Cryo were expanded and mixed with HA/TCP carriers. Calvarial bones, especially parietal bone area (P), were removed to generate a bone defect on immunocompromised mice. SHED & HA/TCP mixture were implanted to cover over the defect area. Twelve weeks after the implantation, the samples were harvested and analyzed by microCT and histology. (PDF) [file pone.0051777.s008.pdf]
