# Supplementary material for: Cryopreserved Dental Pulp Tissues of Exfoliated Deciduous Teeth Is a Feasible Stem Cell Resource for Regenerative Medicine
Source: PLoS One. 2012 Dec 14;7(12):e51777. doi: 10.1371/journal.pone.0051777 (PMC3522596; doi:10.1371/journal.pone.0051777)
Supplement: Table S1 — The list of antibodies. (PDF) [file pone.0051777.s009.pdf]

Table S1.

Names of antibodies	Types of antibodies	Names of Suppliers
anti-CD3 antibody	purified	eBioscience (San Diego, CA)
anti-CD4 antibody	R-PE-conjugated	eBioscience (San Diego, CA)
anti-CD8a antibody	FITC-conjugated	eBioscience (San Diego, CA)
anti-CD14 antibody	R-PE -conjugated	eBioscience (San Diego, CA)
anti-CD28 antibody	purified	eBioscience (San Diego, CA)
anti-CD31 antibody	purified	eBioscience (San Diego, CA)
anti-CD34 antibody	R-PE-conjugated	eBioscience (San Diego, CA)
anti-CD45 antibody	R-PE-conjugated	eBioscience (San Diego, CA)
anti-CD73 antibody	R-PE-conjugated	eBioscience (San Diego, CA)
anti-CD90 antibody	R-PE-conjugated	eBioscience (San Diego, CA)
anti-CD105 antibody	R-PE-conjugated	eBioscience (San Diego, CA)
anti-CD146 antibody	R-PE-conjugated	eBioscience (San Diego, CA)
anti-interferon γ antibody	APC-conjugated	eBioscience (San Diego, CA)
anti-interleukin 17A antibody	R-PE-conjugated	eBioscience (San Diego, CA)
anti-mitochondria human specific	purified	Millipore (Billerica, MA)
anti-nestin antibody	R-PE-conjugated	BD Biosciences (San Jose, CA)
anti-neurofilament M antibody	purified	Sigma (St Louis, MO)
anti-STRO-1 antibody	purified	abcam (Cambridge, MA)
anti-tubulin βIII antibody	purified	Sigma (St Louis, MO)

APC: allophycocyanin

FITC: fluorescein isothiocyanate

PerCP: Peridinin-chlorophyll-protein complex

R-PE: R-phycoerythrin
