# Supplementary material for: Cryopreserved Dental Pulp Tissues of Exfoliated Deciduous Teeth Is a Feasible Stem Cell Resource for Regenerative Medicine
Source: PLoS One. 2012 Dec 14;7(12):e51777. doi: 10.1371/journal.pone.0051777 (PMC3522596; doi:10.1371/journal.pone.0051777)
Supplement: Table S2 — The list of primer pairs for RT-PCR. (PDF) [file pone.0051777.s010.pdf]

Table S2.

albumin (GenBank accession no. NM_000477.5)
sense: 5'-ATGGATGATTTCGCAGCTTT-3' (1787-1806)
antisense: 5'- TGGCTTTACACCAACGAAAA-3' (2005-1986)

aggrecan (NM_013227)
sense: 5'-GCAGAGACGCATCTAGAAATTG-3' (7062-7083)
antisense: 5'-GGTAATTGCAGGGAACATCATT-3' (7656-7677)

alkaline phosphatase (*ALP*) (X14390)
sense: 5’-ACGTGGCTAAGAATGTCATC-3’ (nucleotide 322-341)
antisense: 5’-CTGGTAGGCGATGTCCTTA-3’ (nucleotide 779-797)

collagen, type X (*ColX*) (NM_000493)
sense: 5'-CACCAGGCATTCCAGGATTCC-3' (1463-1483)
antisense: 5'-AGGTTTGTTGGTCTGATAGCTC-3'(2265-2286)

dentin sialophosphoprotein (*DSPP*) (NM_014208)
sense: 5′-GGCAGTGACTCAAAAGGAGC-3′ (1630-1649)
antisense: 5′-TGCTGTCACTGTCACTGCTG-3′ (1815-1834)

glyceraldehyde 3-phosphate dehydrogenase (*GAPDH*) (M33197)
sense: 5′-AGCCGCATCTTCTTTTGCGTC-3′ (12-32)
antisense: 5′-TCATATTTGGCAGGTTTTTCT-3′ (807-827)

lipoprotein lipase (*LPL*) (X14390)
sense: 5′-ATGGAGAGCAAAGCCCTGCTC-3′ (118-138)
antisense: 5′-GTTAGGTCCAGCTGGATCGAG-3′ (661-681)

low-affinity neural growth factor (*LNGFR*) (NM_002507)
sense: 5’-CACCTCCAGAACAAGACCTC-3’ (775-794)
antisense: 5’-GAGCCGTTGAGAAGCTTCTC-3’ (1167-1186)

NANOG (NM_024865)
sense: 5’-TCCTCCATGGATCTGCTTATTCA-3’ (382-404)
antisense: 5’-CAGGTCTTCACCTGTTTGTAGCTGAG-3’ (616-641)

NESTIN (NM_006617)
sense: 5’-CAGCGTTGGAACAGAGGTTGG-3’ (852-872)
antisense: 5’-TGGCACAGGTGTCTCAAGGGTAG-3’ (1218-1240)

NOTCH1 (NM_017617)
sense: 5’-CACCCAGAACTGCGTGCA-3’ (3840-3857)
antisense: 5’-GGCAGTCAAAGCCGTCGA-3’ (4547-4564)

octamer4 (OCT4) (NM_203289)
sense: 5’-GACAGGGGGAGGGGAGGAGCTAGG-3’ (1495-1518)
antisense: 5’-CTTCCCTCCAACCAGTTGCCCCAAAC-3’ (1613-1638)

osteocalcin (*OCN*) (X53698)
sense: 5′-CATGAGAGCCCTCACA-3′ (18-33)
antisense: 5′-AGAGCGACACCCTAGAC-3′ (316-332)

peroxisome proliferator activated receptor-γ2 (*PPARγ2*) (AB451337)
sense: 5′-CTCCTATTGACCCAGAAAGC-3′ (23-42)
antisense: 5′-GTAGAGCTGAGTCTTCTCAG-3′ (350-369)

runt-related gene 2 (RUNX2) (L40992)
sense: 5′-CAGTTCCCAAGCATTTCATCC-3 (880–900)
antisense: 5′-TCAATATGGTCGCCAAACAG-3′ (1304–1323)

SOX9 (NM_000346)
sense: 5'-GAACGCACATCAAGACGGAG-3' (1553-1572)
antisense: 5'-TCTCGTTGATTTCGCTGCTC-3' (2164-2183).
